# Genomic analyses reveal selection footprints in rice landraces grown under on‐farm conservation conditions during a short‐term period of domestication

**DOI:** 10.1111/eva.12866

**Published:** 2019-09-30

**Authors:** Di Cui, Hongfeng Lu, Cuifeng Tang, Jinmei Li, Xinxiang A, Tengqiong Yu, Xiaoding Ma, Enlai Zhang, Yanjie Wang, Guilan Cao, Furong Xu, Yongli Qiao, Luyuan Dai, Ruiqiang Li, Shilin Tian, Hee‐Jong Koh, Longzhi Han

**Affiliations:** ^1^ National Key Facility for Crop Gene Resources and Genetic Improvement Institute of Crop Sciences Chinese Academy of Agricultural Sciences Beijing China; ^2^ Novogene Bioinformatics Institute Beijing China; ^3^ Institute of Biotech and Germplasm Resources Yunnan Academy of Agricultural Sciences Kunming Yunnan China; ^4^ Department of Plant Science, Plant Genomics and Breeding Institute of Agriculture and Life Science Seoul National University Seoul Korea

**Keywords:** genetic diversity, on‐farm conservation, rice landraces, selection sweep, short‐term domestication

## Abstract

Traditional rice landraces grown under on‐farm conservation conditions by indigenous farmers are extremely important for future crop improvement. However, little is known about how the natural selection and agriculture practices of indigenous farmers interact to shape and change the population genetics of rice landraces grown under on‐farm conservation conditions during the domestication. In this study, we sequenced DNA from 108 core on‐farm conserved rice landraces collected from the ethnic minority regions of Yunnan, China, including 56 accessions collected in 1980 and 52 accessions collected in 2007 and obtained 2,771,245 of credible SNPs. Our findings show that most genetic diversity was retained during the 27 years of domestication by on‐farm conservation. However, SNPs with marked allele frequency differences were found in some genome regions, particularly enriched in genic regions, indicating changes in genic regions may have played a much more prominent role in the short‐term domestication of 27 years. We identified 186 and 183 potential selective‐sweep regions in the *indica* and *japonica* genomes, respectively. We propose that on‐farm conserved rice landraces during the short‐term domestication had a highly polygenic basis with many loci responding to selection rather than a few loci with critical changes in response to selection. Moreover, loci affecting important agronomic traits and biotic or abiotic stress responses have been particularly targeted in selection. A genome‐wide association study identified 90 significant signals for six traits, 13 of which were in regions of selective sweeps. Moreover, we observed a number of significant and interesting associations between loci and environmental factors, which implies adaptation to local environment. Our results provide insights into short‐term evolutionary processes and shed light on the underlying mechanisms of on‐farm conservation.

## 1 INTRODUCTION

Rice (*Oryza sativa* L.) was domesticated between 8,000 and 10,000 years ago from its wild ancestor, *Oryza rufipogon*, a broadly distributed native species of Asia (Oka, 1988). During domestication, its genetic diversity was reduced by up to 80% from that of the wild ancestor (Londo, Chiang, Hung, Chiang, & Schaal, 2006). The most extreme loss of diversity occurred in modern, high‐yield rice cultivars, leading to a lack of evolutionary potential for adaptation to changing environments in these lineages. Compared with modern cultivars, traditional rice landraces grown by indigenous farmers represent an intermediate stage of domestication between a wild ancestor and modern varieties and they serve as reservoirs of genetic variation. These rice landraces are extremely important for future crop improvement because they can be used to breed new cultivars with greater adaptability to biotic and abiotic stress factors (e.g., low moisture, extreme temperatures, disease, pests, and poor soil quality). However, over the past 50 years, local rice landraces have been largely replaced by genetically uniform modern varieties in many parts of China. Rice landraces are no longer planted in the majority of China, except in some ethnic minority regions of Yunnan or Guizhou.

Yunnan Province, located in southwest China (from 21°9′32″N to 29°15′8″N and 97°31′39″E to 106°11′47″E), is characterized by a broad distribution of habitat types, including mountains, plateaus, and basins, with elevations ranging from 76 m to 6,740 m (Zeng et al., 2001). Yunnan has a long history of human settlement and agricultural activities, with 26 nationalities represented in the area. Owing to the wide geographical variation, diverse growing conditions, and cultural and ethnic diversity, Yunnan is acknowledged as one of the largest genetic diversity centers of rice in China and even worldwide (Zeng et al., 2001, 2007; Zhang et al., 2006). A remarkably diverse set of rice landraces are found in Yunnan, including all varieties of *Oryza sativa* L. ssp. *indica* and ssp. *japonica* found in China. The diversity varies in many ways from morphological traits to aromas, such as having glumes with or without hairs and being nonglutinous or glutinous, upland or lowland, of the nude rice type, and rice of various hulled grain colors (white, red, and purple) and flavors (ordinary or fragrant; Zeng, Xu, Shen, & Deng, 2000).

To date, many traditional rice landraces are still planted by indigenous farmers and grown under on‐farm conservation conditions in some ethnic minority regions of Yunnan. They are passed down from generation to generation despite the availability of modern improved varieties for reasons associated with the diversity of the local agroecology and to fulfill cultural requirements (Xu et al., 2014). Indigenous locals practice the conservation of diverse traditional rice landraces on their farms, henceforth referred to as “on‐farm conservation,” not only for the conservation of highly productive landraces but also for ones more resistant to diseases and pests and tolerant of extreme environmental conditions, as well as cultural demands (e.g., ethnic dietary customs, medical uses, festival, and religious ceremony; Gao, 2003). These landraces selected by on‐farm conservation likely cannot be easily replaced by modern improved varieties because they undoubtedly have their own outstanding features and some of these landraces have been planted for more than 50 years. The diverse climatic ecotypes, environmental heterogeneity, and unique ethnic minority cultures and customs play a crucial role in cultivation of landraces by indigenous farmers in the ethnic minority regions of Yunnan and thus provide a model of traditional on‐farm conservation.

On‐farm conservation is a subset of in situ conservation, which is increasingly recognized as a key component of any comprehensive strategy to conserve crop genetic resources (Pandey, Bisht, Bhat, & Mehta, 2011; Pusadee, Jamjod, Chiang, Rerkasem, & Schaal, 2009). Bellon, Pham, and Jackson (1997) defined on‐farm conservation of crop genetic resources as “the continued cultivation and management of a diverse set of traditional landraces by farmers in the agroecosystem where they were developed,” which prevents the traditional landraces from being replaced by modern varieties or disappearing. Thus, on‐farm conservation provides opportunities for continuous differentiation and variation in traditional landraces (Bellon et al., 1997).

Genetic variation and differentiation are influenced by natural processes, such as selection and drift, and can also be influenced by the agriculture practices of indigenous farmers. However, little is known about how these processes interact to shape and change the population genetics of rice landraces grown under on‐farm conservation conditions during a period of short‐term domestication, which we consider as less than 30 years. Similarly, it is not clear whether genetic diversity has been successfully maintained by on‐farm conservation practices. Thus, we aim to use whole‐genome sequences to address how population dynamics have changed or remained the same over a short period of domestication in rice landraces cultivated following on‐farm conservation practices. Characterization of genome‐wide selection footprints and genetic diversity may help reveal the short‐term evolutionary processes and contribute to our understanding of the mechanisms of on‐farm conservation.

## 2 MATERIALS AND METHODS

### 2.1 Sample collection

We performed a large scale study using 600 on‐farm conserved rice landraces, including 332 accessions collected in 1980 and 268 accessions collected in 2007 from the ethnic minority regions of Yunnan Province, China, in our previous study (Cui et al., 2016). These accessions were from five different ecological zones, covering a wide geographic distribution and diverse growing conditions, and represent most of the diversity of on‐farm conserved landraces in Yunnan. In this study, a core subset of the collection of 600 landraces (108 accessions, including 56 accessions collected in 1980, and 52 accessions collected in 2007; Table S1 and Figure S1) was sampled for whole‐genome sequencing and construction of neighbor‐joining (NJ) trees using 48 SSRs (Table S2) that represented more than 97% of the genetic diversity of a total of 600 accessions in the DNA information obtained (Figure S2).

### 2.2 DNA sequencing and mapping

To construct the DNA libraries, genomic DNA from the fresh leaves of each of the 108 landraces was extracted using the CTAB method (Murray & Thompson, 1980). All the libraries were sequenced by the high‐throughput Illumina sequencing platform (HiSeq 2500) using standard procedures of Novogene Bioinformatics Institute (Beijing, China). The 500‐bp paired‐end libraries were constructed according to the manufacturer's introductions (Illumina). Using a whole‐genome shotgun strategy, we generated a total of 2.28 Gb of paired‐end reads of 125‐bp length (284.77 Gb of sequences). Prior to mapping, all reads were preprocessed for quality control and filtered out using our in‐house script in PERL according to the following criteria: (a) any reads with ≥10% unidentified nucleotides (N); (b) any reads with >10 nt aligned to the adapter sequence, allowing ≤10% mismatches; (c) any reads with >50% bases having phred quality <5; and (d) putative PCR duplicates generated by PCR amplification in the library construction process (i.e., two paired‐end reads were the same). A total of 280.66 Gb high‐quality sequences was kept and mapped to the rice reference genome (ftp://ftp.ensemblgenomes.org/pub/plants/release-23/fasta/oryza_sativa/dna/Oryza_sativa.IRGSP-1.0.23.dna_sm.toplevel.fa.gz) using BWA‐MEM with default parameters except for the “‐t ‐k 32 ‐M ‐R” option (Li & Durbin, 2009). Alignment bam files were sorted using SAMtools (Li et al., 2009), and duplicated reads were removed. Sequencing coverage and depth for each sample were calculated, and the average sequencing coverage was 88.65% (Table S3). All genome sequence data have been deposited in the NCBI Sequence Read Archive under project accession number: PRJNA342109.

### 2.3 Detection of variation

We performed variation calling for the 108 accessions using a Bayesian approach implemented in the package SAMtools (Li *et al.*, 2009b). The “mpileup” command was used to identify SNPs and Indels with the parameters “‐m 2 –F 0.002 ‐d 1,000.” In the downstream analysis, the raw population variations were filtered by requiring a respective minimum and maximum coverage depth of 4 and 1,000, a minimum RMS (root mean square) mapping quality score of 20, and missing genotype >50% of the 108 rice accessions. Consequently, a total of 2,771,245 of SNPs and 432,174 indels (204,215 insertions and 227,959 deletions, ranging from 1 to 5 bp in length) were retained for downstream analyses (Tables S4–S5). Finally, all of the genomic variations were annotated using ANNOVAR software (Wang, Li, & Hakonarson, 2005).

### 2.4 Population analysis

#### 2.4.1 Population structure

To estimate individual admixture assuming different numbers of clusters, the population structure was investigated using ADMIXTURE (Alexander, Novembre, & Lange, 2009) with a maximum likelihood method. We increased the coancestry clusters spanning from 2 to 8 and ran the analysis with 10,000 iterations (Figure S3). The optimal k‐value was determined based on cross‐validation error (Figure S4).

#### 2.4.2 Principal components analysis

The software package GCTA (Yang, Lee, Goddard, & Visscher, 2011) was used for principal component analysis with biallelic SNPs of the 108 individuals. We plotted the first two significant components. To some extent, the discrete points reflect the real structure of population.

#### 2.4.3 Phylogenetic tree

We constructed a neighbor‐joining tree using a matrix of pairwise genetic distances of all individuals, calculated by TreeBest (http://treesoft.sourceforge.net/treebest.shtml), which first runs a number of independent phylogenetic methods and then creates a combined tree using a stochastic context ‐free grammar approach. The bootstrap test (Efron 1982; Felsenstein, 1985) was used for evaluating the reliability of a neighbor‐joining tree. For this method, the same number of sites was randomly sampled with replacement from the original sequences, and a phylogenetic tree was constructed from the resampled data. This process was repeated, and the reliability of a sequence cluster was evaluated by its relative frequency of the appearance in bootstrap replications (Kumar, Tamura, & Nei, 1994). The bootstrap was set to 1,000 times to assess the branch reliability using TreeBest.

### 2.5 Selection analyses

A sliding‐window approach (10‐kb windows sliding in 1‐kb steps) was applied to quantify polymorphism levels (*θ*_π_, pairwise nucleotide variation as a measure of variability) and genetic differentiation (*F*_ST_) between rice landraces from 1980 to 2007 using VCFtools (Danecek et al., 2011).

To detect regions with significant signatures of selective sweep, we Z‐transformed the distribution of *F*_ST_ and calculated the log value of *θ*_π_ ratios (*θ*_π 1980_/*θ*_π 2007_). We used an empirical procedure and selected windows with significantly high log_2_ (*θ*_π 1980_/*θ*_π 2007_) and Z*F_ST_* values from their respective empirical distributions and considered these as regions with selective‐sweep signals along the genome. The values were at the right 5% tails, where the log_2_ (*θ*_π 1980_/*θ*_π 2007_) thresholds were 1.02 and 1.41 for *indica* and *japonica*, respectively, and Z*F_ST_* thresholds were 1.92 and 2.00, respectively. Then, adjacent selective‐sweep regions were merged together to form a whole putative selected region. To further confirm the selection signals identified, we performed a genome scan using a cross‐population composite likelihood approach XP‐CLR (Chen, Patterso, & Reich, 2010) to calculate the candidate selective‐sweep regions. A 10‐kb sliding window with 1‐kb steps across the whole genome was used for scanning. The highest XP‐CLR values, accounting for 5% of the genome (*p* < .05), were considered as selected regions.

### 2.6 Annotation analysis of selected regions

We annotated genes in selected genomic regions using the rice genome and a total of 623 and 537 genes in *indica* and *japonica*, respectively. They were identified to undergo strong selection sweep. These genes were submitted to Gene Ontology (GO) (Ashburner et al., 2000) and Kyoto Encyclopedia of Genes and Genomes (KEGG) (Kanehisa & Goto, 2000) databases for enrichment analyses. A false discovery rate (FDR)‐corrected binomial distribution probability approach was used to test significant enriched gene function at a level of *p* < .05 (Benjamini & Hochberg, 1995).

### 2.7 Genome‐wide association study of agronomic traits

We performed a genome‐wide association study (GWAS) for ten agronomic traits: days to heading, plant height, panicle length, effective panicles, grains per panicle, grain length, grain width, grain length‐to‐width ratio, spikelet fertility, and 1,000‐grain weight. All landraces were planted on November 27, 2011, in the Hainan experimental station of Chinese Academy of Agricultural Sciences and July 16, 2012, in the Xishuangbanna experimental station of Yunnan Academy of Agricultural Sciences. Phenotypic measurements of the traits were recorded from the ten middle plants, and mean values of traits were calculated for each landrace. Finally, the average phenotypic value of two environments was used for the GWAS.

Association analyses were conducted using MLMs (Yu et al., 2006) with TASSEL v.5.0 (http://www.maizegenetics.net/tassel). A kinship matrix (K‐matrix), the pairwise relationship matrix calculated by TASSEL v.5.0, and the Q‐matrix as a correction for population structure were used in the MLM association models to calculate P‐values to associate each SNP marker with the trait of interest and to avoid spurious associations by TASSEL v.5.0.

### 2.8 Environmental association analysis

Latent factor mixed models (LFMM) (Frichot, Schoville, Guillaume, & Francois, 2013) were used to identify genetic variants associated with 22 particular environmental factors (including elevation, monthly maximum/minimum temperature, and monthly precipitation from April to October of the rice ‐growing season) for all samples collected in both 1980 and 2007. Climate data were obtained from NMC (National Meteorological Center) in China (http://www.nmc.cn/). The K‐value was set to 2 based on the eigenvalues of the PCA of the genetic data as the number of latent factors. Five replicates were verified for convergence. The median z‐scores of five runs were used to re‐adjust the p‐values. For an expected value of the FDR (*q* = 5%), a list of candidate loci were obtained by using the Benjamini–Hochberg procedure.

## 3 RESULTS

### 3.1 Genomic variation

We generated 280.66 Gb of high‐quality sequences from 108 on‐farm conserved rice landraces, which consisted of 56 accessions collected in 1980 and 52 accessions collected in 2007 (Table S1 and Figure S1). After alignment, 97.17% of the reads covered 88.65% of the reference genome, with an average of 6.94‐fold depth (Table S3). We subsequently identified 2,771,245 credible SNPs from the 108 accessions. Among all SNPs, 1,935,246 (69.83%) were located in intergenic regions and only 171,050 (6.17%) were located in coding regions (Table S4). The ratio of nonsynonymous to synonymous substitutions of the SNPs was calculated to be 1.20, which is consistent with previous reports (Xu et al., 2012). We observed low heterozygosity in all accessions, reflecting the lack of cross‐pollination owing to high levels of inbreeding (Figure S5).

In addition, a total of 204,215 insertions and 227,959 deletions were identified, ranging from 1 to 5 bp in length (Figure S6). We annotated 9,000 (2.08%) indels located in coding regions of the rice genome. Of these, 4,044 indels could have large effects on the protein‐coding sequences, including 3,930 indels that cause frameshift changes, 78 indels that lead to the immediate creation of a stop codon, and 36 indels that lead to the immediate elimination of a stop codon (Table S5).

### 3.2 Population structure and genetic diversity

**Figure 1:**
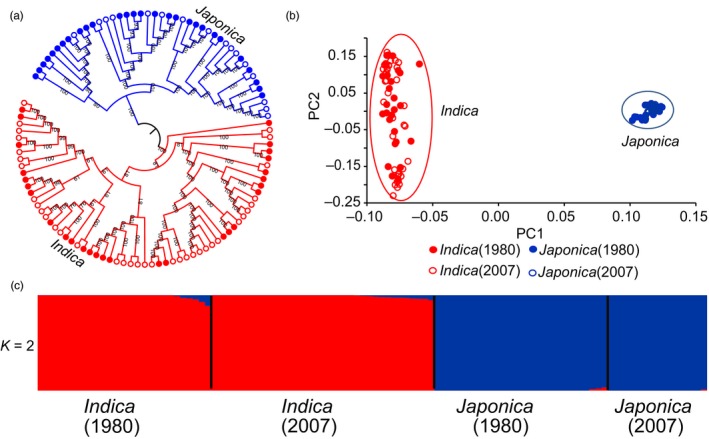
Population structure analysis of 108 rice landraces collected in 1980 and 2007. (a) NJ phylogenetic tree inferred from whole‐genome SNPs. Bootstrap values (>50) are shown on each branch. (b) PCA plots of the first two components of 108 accessions. (c) Population structure inferred using ADMIXTURE with *K* = 2. The length of each segment in each vertical bar represents the proportion contributed by ancestral populations

We explored phylogenetic relationships among the 108 rice landraces through whole‐genome SNP analysis (Figure 1a). As expected, the phylogenetic tree showed that all the rice landraces were clearly divided into two major groups of cultivated rice, 64 into the *indica* group and 44 into the *japonica* group. In each group, there were rice landraces from both collections in 1980 and 2007 (Figure 1a). These results were further supported by the principal component analysis and structural analysis (Figure 1b, 1c). In a comparison of the *indica* landraces collected in 1980 and 2007, the two sets of *indica* landraces separated by approximately 27 years of domestication exhibited very similar genetic diversity levels (*θ*_π_ = 1.21 × 10^–3^ in 1980, *θ*_π_ = 1.20 × 10^–3^ in 2007) (Table 1) and showed little genetic differentiation (*F*_ST_ = 0.026). A similar trend in genetic diversity was found in *japonica* (*θ*_π_ = 7.47 × 10^–4^ in 1980; *θ*_π_ = 6.80 × 10^–4^ in 2007; *F*_ST_ = 0.036). This result indicates that most genetic diversity in both *indica* and *japonica* landrace germplasms grown under on‐farm conservation conditions was retained during the short‐term period of domestication.

**Table 1:** Genetic diversity in whole‐genome, genic, and intergenic regions of rice landraces in 1980 and 2007

Group	Year	Whole‐genome		Genic region		Intergenic region	
		*θ*_π_ (10^–3^)	*F*_ST_	*θ*_π_ (10^–3^)	*F*_ST_	*θ*_π_ (10^–3^)	*F*_ST_
Indica	1980	1.205	0.026	0.311	0.031	1.035	0.026
	2007	1.199		0.315		1.028	
Japonica	1980	0.747	0.036	0.211	0.043	0.643	0.037
	2007	0.680		0.193		0.586	

**Figure 2:**
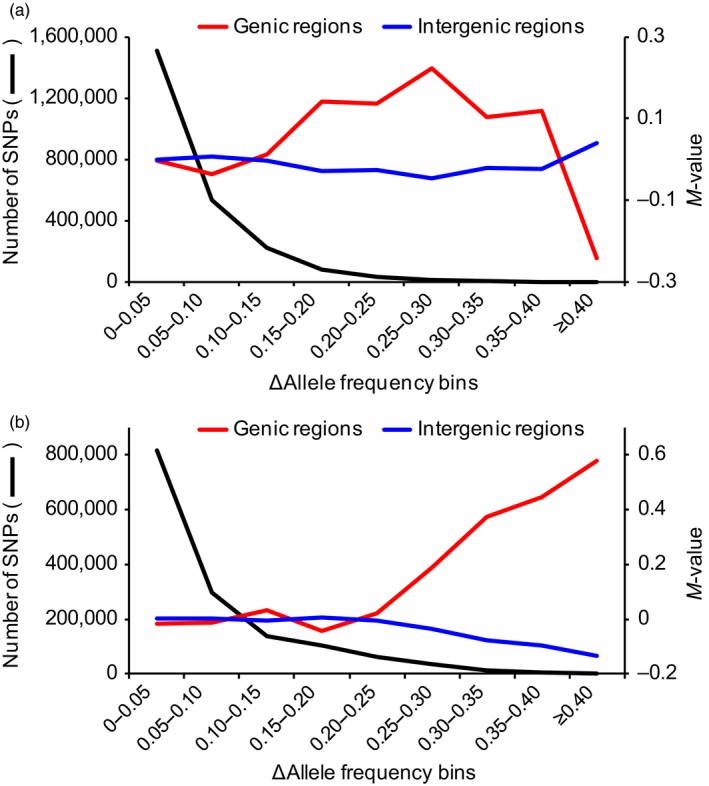
The majority of SNPs showed low ΔAF between rice landraces collected in 1980 and 2007. (a) and (b) show *indica* and *japonica* landraces, respectively. The black line indicates the number of SNPs in nonoverlapping ΔAF bins (left y‐axis). Colored lines denote M values (log2‐fold changes) of the relative frequencies of SNPs at genic (red) and intergenic regions (blue), according to ΔAF bins (right y‐axis). M values were calculated by comparing the frequency of SNPs in a given annotation category in a specific bin with the corresponding frequency across all bins

Based on the correlation in allele frequencies between landraces collected in the two years, for each SNP, we calculated the absolute allele frequency difference (ΔAF) between *indica* or *japonica* rice collected in 1980 and 2007 and sorted these into 5% bins (ΔAF = 0 to 0.05, etc.; Figure 2 and Tables S6–S7). For both *indica* and *japonica*, the majority of SNPs showed low ΔAF between landraces from 1980 to 2007 (Figure 2 and Tables S6–S7). Only 0.42% of total SNPs in *indica* and 1.31% of total SNPs in *japonica* with ΔAF ≥ 0.30 were highly differentiated (Tables S6–S7). We further examined genic and intergenic regions for enrichment of SNPs with high ΔAF, which is expected under directional selection on many independent mutations. In *indica*, we observed no enrichment for SNPs with high ΔAF in intergenic regions, but we found a significant excess of SNPs in genic regions with ΔAF of 0.30–0.35 (chi‐square test, *p* = 3.91 × 10^–2^; Table S6). In *japonica*, we detected a significant excess of SNPs in genic regions for each bin of ΔAF ≥ 0.30 ( chi‐square test, *p* = 2.43 × 10^–13^ to *p* = 2.60 × 10^–41^), but no excess of SNPs with high ΔAF in intergenic regions (Table S7). Compared to the relative proportions in the entire data set, there was an excess of SNPs (58 SNPs) in genic regions with ΔAF of 0.30–0.35 in *indica* and an excess of 949 SNPs with ΔAF ≥ 0.30 in *japonica* (Tables S6–S7).

### 3.3 Genomic imprints of selection in rice landraces

**Figure 3:**
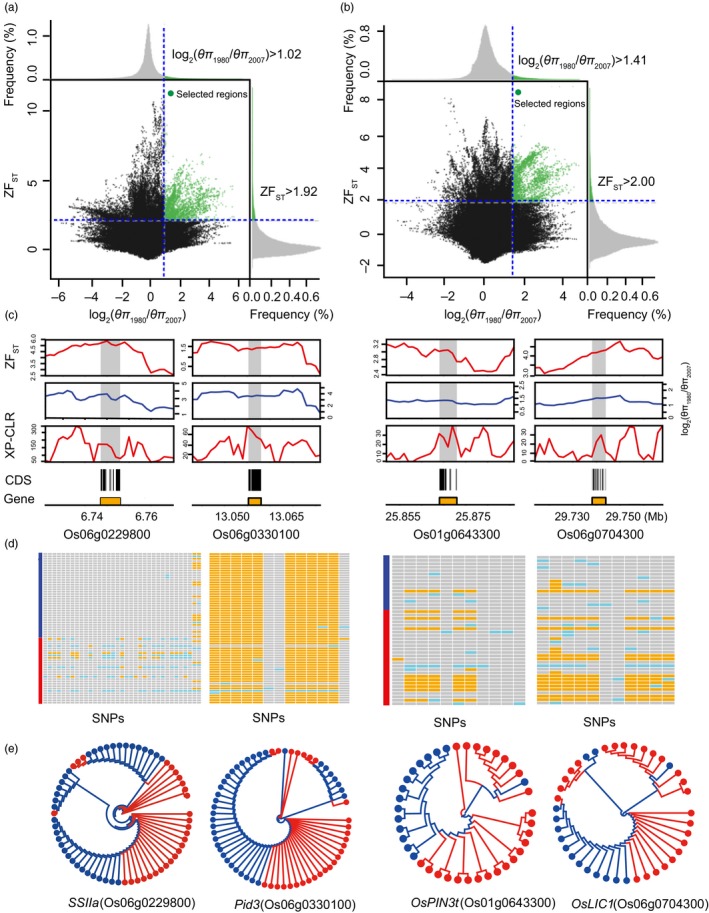
Genomic regions with strong selective‐sweep signals in rice landraces grown under on‐farm conservation conditions during a short‐term period of domestication. (a, b) Distribution of log_2_ (*θ*_π 1980_/*θ*_π 2007_) and Z*F*_ST_ values, which were calculated in 10‐kb windows sliding in 1‐kb steps. Vertical dashed lines correspond to the right 5% tail of the empirical log_2_ (*θ*_π 1980_/*θ*_π 2007_) distribution, where the log_2_ (*θ*_π 1980_/*θ*_π 2007_) values are 1.02 and 1.41 in *indica* (a) and *japonica* (b), respectively, and horizontal dashed lines correspond to the right 5% tail of the empirical Z*F*_ST_ distribution, where the Z*F*_ST_ values are 1.92 and 2.00 in *indica* (a) and *japonica* (b), respectively. Data points located to the right of the vertical dashed lines and above the horizontal dashed lines were identified as strong selective‐sweep regions for *indica* and *japonica* landraces (green points). (c) Example of genes in genomic regions with strong selective‐sweep signals. log_2_ (*θ*_π 1980_/*θ*_π 2007_), Z*F*_ST_ values and XP‐CLR values of landraces plotted using a 10‐kb sliding window. Genomic regions located simultaneously with significantly high values of each log_2_ (*θ*_π 1980_/*θ*_π 2007_) (5% right tail), Z*F*_ST_ (5% right tail) and XP‐CLR (5% right tail) were considered as regions with strong selective‐sweep signals (gray regions). Genome annotations are shown at the bottom (black bar, coding sequences (CDS); color bar, gene). (d) Status of SNPs per line with the reference allele in gray, homozygous SNPs in orange and heterozygous SNPs in light blue. (e) Gene trees are for *SSIIa* (Os06g0229800), *Pid3* (Os06g0330100), *OsPIN3t* (Os01g0643300), and *OsLIC1* (Os06g0704300) of the landraces collected in 1980 (red) and 2007 (blue)

**Figure 4:**
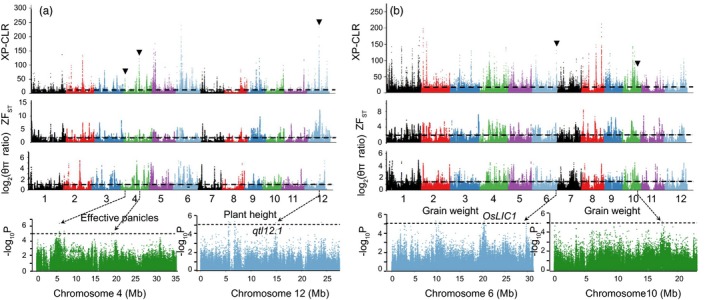
GWAS results for three traits that had overlapping selective‐sweep regions in *indica* and *japonica* genomes during short‐term domestication. (a) and (b) show the whole‐genome screening of selective‐sweep signals in *indica* and *japonica* genomes, respectively. The XP‐CLR value, log_2_ (*θ*_π_ ratio [*θ*_π 1980_/*θ*_π 2007_]) and Z*F*_ST_ values are plotted against the position on each of the 12 chromosomes. The horizontal dashed lines indicate the genome‐wide threshold of selective‐sweep signals. (c) The four GWAS results that overlapped strong selective‐sweep signals. The horizontal dashed lines indicate the significance threshold of GWAS (–log_10_ *p* > 5)

To more accurately detect the genomic footprints of farmer selection in combination with natural selection during the 27‐year domestication period, we measured genome‐wide variation between rice landraces from 1980 to 2007. To reduce the impact of genetic divergence between subspecies of *indica* and *japonica* landraces, we calculated the nucleotide diversity ratio (*θ*_π 1980_/*θ*_π 2007_) and genetic differentiation (*F*_ST_) between rice landraces in 1980 and 2007 for sliding windows in both *indica* and *japonica* landraces, respectively (Figures S7–S8). Using the top 5% of log_2_ (*θ*_π 1980_/*θ*_π 2007_) values, we identified 891 and 954 candidate regions with selective‐sweep signals (Figure 3a, b and Figures S7–S8) in *indica* and *japonica* landraces, respectively. Notably, 84 common regions underwent selective sweeps in both *indica* and *japonica*, indicating these regions might be commonly selected in both subspecies during the 27‐year domestication period. Alternatively, they could be subspecies‐specific selective‐sweep regions that were independently selected in either subspecies (Xu et al., 2012). Using the top 5% of Z*F*_ST_ values, we also detected 677, 790 and 107 candidate regions in *indica*, *japonica*, and both subspecies, respectively (Figure 3a, 3b and Figures S7–S8). Strikingly, we identified 186 potential selective‐sweep regions with an average size of 27.08 kb, comprising approximately 5.04 Mb or 1.35% of the assembled genome of *indica*, and 183 potential selective‐sweep regions with an average size of 26.81 kb, comprising approximately 4.91 Mb or 1.31% of the assembled genome of *japonica* based on both methods (Figure 3a, 3b and Tables S8–S9). Moreover, results of both methods indicate that only two common regions underwent selective sweeps in both *indica* and *japonica*. To further confirm these selection signals identified above, we performed a genome scan using a cross‐population composite likelihood approach XP‐CLR (Chen et al., 2010) to calculate the candidate selective‐sweep regions. We found that more than 90% of the selective‐sweep regions overlapped with genomic regions identified as showing selective sweeps by the XP‐CLR approach (Figure 4a, b), indicating that most of the selection regions can be supported by the XP‐CLR approach and is thus quite reliable. These regions exhibited significant differences (*p* < 10^–15^, Mann–Whitney U test) in log_2_ (*θ*_π 1980_/*θ*_π 2007_) and Z*F*_ST_ values compared with the genomic background in both *indica* and *japonica* (Figure S9). These regions also had lower levels of nucleotide diversity and extremely negative Tajima's D‐values (Table S10). This result quantitatively reflects the importance of selection in shaping genomic variation, resulting in phenotypic and/or environmental adaptations in rice landraces. In the selective‐sweep regions, we identified 623 and 536 protein‐coding genes in the respective *indica* and *japonica* genomes, which are expected to represent targets of selection. To assess the functions of these candidate genes, we used GO (Ashburner et al., 2000) and KEGG (Kanehisa & Goto, 2000) functional categories based on orthologs to annotate them. We found that functional categories related to morphology, growth and development, transcriptional regulation, and metabolic processes were enriched (Tables S11–S14).

### 3.4 Selective‐sweep regions included important agronomic genes

**Figure 5:**
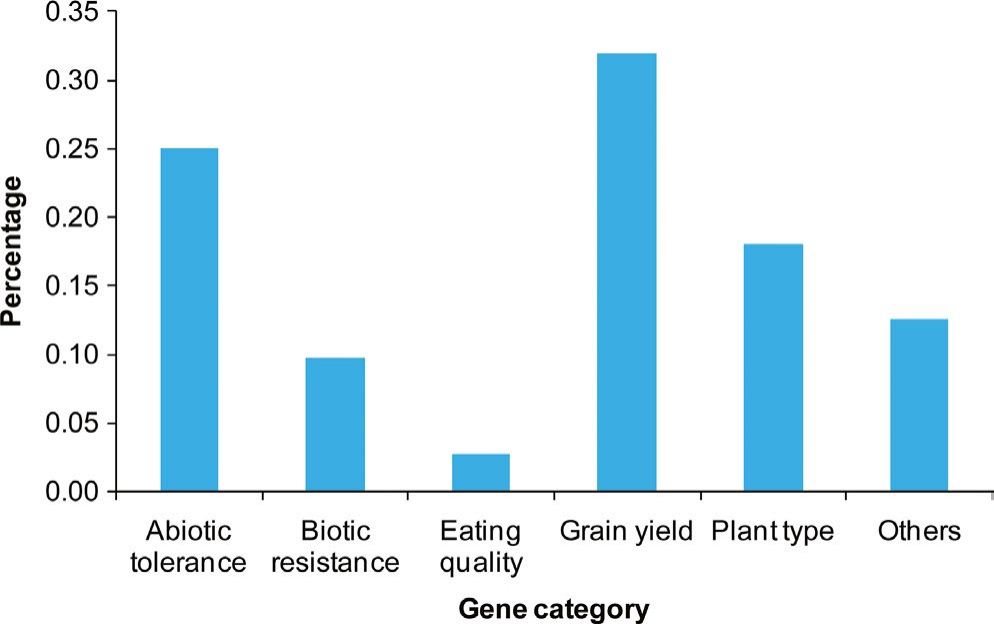
Functional category of cloned genes in selective‐sweep regions

As expected, we found a number of candidate genes included in the potential selective‐sweep regions were important agronomic genes/quantitative trait loci (QTLs) released by Q‐TARO (Yonemaru, Yonemaru, Yamamoto, & Yano, 2012) (Tables S15–S17). For example, a strong signature of selection (log_2_ [*θ*_π 1980_/*θ*_π 2007_] = 2.29, Z*F*_ST_ = 3.55, XP‐CLR = 144.28) was observed for a region on chromosome 6 of the *indica* genome, which includes the *SSIIa* (Similar to Starch synthase IIA) gene (Figure 3c). This gene is responsible for the eating quality of rice and plays an important role in grain starch synthesis (Kawakatsu, Yamamoto, Touno, Yasuda, & Takaiwa, 2009; Zhang et al., 2011). Patterns at *SSIIa* revealed reduced diversity in the rice landraces collected in 2007 when compared with diversity of that collected in 1980 (Figure 3d). In each clade of the *SSIIa* tree, we found most landraces collected in the same year clustered together, thus indicating a tendency of differentiation between landraces collected in 1980 and 2007 (Figure 3e). This result may also provide evidence for a potential selective‐sweep region. Two of the other selective‐sweep candidates contained major genes related to biotic stress in rice. One candidate, located at 13.045–13.060 Mb on chromosome 6 (log_2_ [*θ*_π 1980_/*θ*_π 2007_] = 3.69, Z*F*_ST_ = 2.01, XP‐CLR = 39.35), encompassed the entire coding region of *Pid3* (*Pyricularia oryzae resistance‐d3*), which confers blast resistance to *Magnaporthe oryzae* (Chen et al., 2011; Shang et al., 2009; Xu et al., 2014); the other candidate, located at 8.075–8.114 Mb on chromosome 11, includes *NLS1*, which encodes a typical CC‐NB‐LRR‐type protein and is related to resistance to bacterial pathogens (Tang et al., 2011). Meanwhile, the region on chromosome 1 containing *OsPIN3t* showed a selective‐sweep signal (log_2_ [*θ*_π 1980_/*θ*_π 2007_] = 1.77, Z*F*_ST_ = 5.50, XP‐CLR = 28.51) in the *japonica* genome and a *SSIIa*‐like gene tree (Figure 3c and 3e). This gene is involved in drought stress response and drought tolerance (Miyashita, Takasugi, & Ito, 2010; Wang et al., 2009; Zhang et al., 2012). Additionally, a selection signal (log_2_ [*θ*_π 1980_/*θ*_π 2007_] = 2.29, Z*F*_ST_ = 3.55, XP‐CLR = 19.28) was detected in a region on chromosome 6 of the *japonica* genome, which includes the *OsLIC1* gene (Figure 3c). This gene is involved in the regulation of rice plant architecture and grain yield (Wang et al., 2008; Zhang et al., 2012). In total, we found 72 cloned agronomic genes in selective‐sweep regions of the *indica* and *japonica* genomes. Notably, these selective‐sweep regions were extremely enriched in grain yield and abiotic stress resistance gene categories, followed by the plant‐type category (Figure 5). Furthermore, nearly all of our selective‐sweep regions were overlapped with previously reported agronomic QTLs (Tables S16–17). However, the sizes of the selective‐sweep regions were smaller than sizes of the reported QTLs (Tables S16–S17), which indicates that these defined regions will be helpful in identifying genes that govern important agronomic traits.

To further annotate the selective‐sweep regions, we performed GWAS for ten agronomic traits (Table S18 and Figures S10–S12). In total, we identified 90 significant signals for six traits, some of which were also mapped for the same trait in previous studies (Table S18). GWAS signals associated with 1,000‐grain weight and effective panicles were detected at the previously reported *qyd1* locus on Chr. 1 and the *qHbd3* locus on Chr. 11 (Table S18; Yonemaru et al., 2012), respectively. We also detected a GWAS signal responsible for plant height at the *qtl12.1* locus (Figure 4a, c) and a GWAS signal corresponding to 1,000‐grain weight at the *OsLIC1* locus (Figure 4b, c) (Yonemaru et al., 2012). We found that these two signals underwent selection during the short‐term domestication of *indica* and *japonica*, respectively (Figure 4). Notably, the strongest GWAS signal responsible for effective panicles overlapped with a selective sweep originating on Chr. 4 during the short‐term domestication of *indica* (Figure 4a, c). In total, we identified 13 GWAS signals overlapped with the selective sweeps during short‐term domestication (Table S18).

### 3.5 Genomic signatures of local adaptation

To screen genomes for signatures of local adaptation, we tested for associations between genetic variation and environmental gradients using latent factor mixed models (LFMM) (Frichot *et al.*, 2012). A total of 986 SNPs that obtained |z|‐scores greater than 5 (*p* < 10^–10^) (Table S19) were associated with elevation. Among these SNPs, we found the SNP (|z| = 5.25) located at 29.62 Mb on chromosome 7 was significantly associated with elevation. Interestingly, this SNP was in the gene region of *DTH7* involving in heading date in rice (Gao et al., 2014; Koo et al., 2013; Liu, Liu, Zhang, & Xing, 2013), which indicated this candidate gene might be related to adaptation to elevation (Table S20). We also found a significant SNP (|z| = 5.48) on chromosome 9 in the gene *OsWRKY62* (Table S20), which is involved in bacterial blight resistance (Peng et al., 2008), reflecting its adaption to the elevation. In addition, we found a total of 938 and 1,085 SNPs that obtained |z|‐scores greater than 5 (*p* < 10^–10^) were associated with monthly temperature and precipitation, respectively (Tables S21–S22). Among these SNPs significantly correlated with climatic gradients, several notable examples include candidate genes involved in grain shape (*GW7* and *CYP78A13*; Nagasawa et al., 2013; Wang et al., 2015), plant height (*OsCYP96B4* and *SLRL1*; Fukao & Bailey‐Serres 2008; Itoh et al., 2005; Ramamoorthy, Jiang, & Ramachandran, 2011), eating quality (*OsBEIIb*; Tanaka et al., 2004), bacterial blight resistance (*OsWRKY62*; Peng et al., 2008), cold tolerance (*Ctb1*; Saito, Hayano‐Saito, Kuroki, & Sato, 2010; Saito, Hayano‐Saito, Maruyama‐Funatsuki, Sato, & Kato, 2004), etc. (Table S20). All of these significant associations between loci/genes and environmental variables imply adaptation to the local environment. In total, in terms of the loci with high levels of association with environmental variables, we found 63 loci were also in the selective‐sweep regions (Table S23), which indicates that local adaptation might play an important role in short‐term domestication.

## 4 DISCUSSION

To date, few studies have focused on the short‐term domestication process and the population dynamics underlying short‐term domestication are still poorly understood (Cui et al., 2016; Sun, Cao, Ma, Chen, & Han, 2012; Xu et al.., 2011; Yan et al.., 2012). This study used genome sequencing data (including millions of polymorphisms) to provide an unprecedented opportunity to comprehensively identify and characterize population dynamics in on‐farm conserved samples of rice genetic resources and to reveal the selection footprints in rice landraces during the short‐term domestication process.

Rice (*Oryza sativa* L.) was domesticated from wild rice (*Oryza rufipogon*) thousands of years ago (Oka, 1988). During this long‐term period of domestication, its genetic diversity was reduced by up to 80% from the wild ancestor due to strong selection and genetic bottlenecks (Londo et al., 2006). Relative to long‐term domestication, our short‐term domestication study comparing representative on‐farm conserved landraces collected in 1980 to the landraces collected in 2007 resulted in similar levels of genome‐wide genetic diversity both in *indica* and *japonica* landraces. Therefore, on‐farm conservation conditions successfully maintained genetic diversity during at least the 27 years of domestication. This result was consistent with our previous study (Cui et al., 2016; Li et al., 2015). Similar results were found in a study of sorghum (Deu et al., 2010) in which crop management by farmers globally preserved sorghum genetic diversity in Niger over a 26‐year period (1976–2003). We further calculated the ΔAF between *indica* or *japonica* rice landraces collected in 1980 and 2007, and examined genic and intergenic regions for enrichment of SNPs with high ΔAF. Although the majority of SNPs showed low ΔAF between *indica* or *japonica* rice landraces collected in 1980 and 2007, it is noteworthy that some highly differentiated individual SNPs (ΔAF ≥ 0.30) were found in *indica* and *japonica* genomes, which were likely directly targeted by selection during the 27‐year domestication period. Notably, we found that SNPs with marked allele frequency differences between rice landraces collected in 1980 and 2007 were enriched in genic regions, which suggests changes in genic regions may have played a much more prominent role in short‐term domestication than changes in intergenic regions.

Next, we considered the potential role of farmer selection in combination with natural selection in affecting rice landraces grown under on‐farm conservation conditions during the 27 years of domestication. We performed a genome‐wide scan for signatures of selective sweeps, and identified 186 and 183 potential selective‐sweep regions (containing 623 and 526 candidate genes) in the *indica* and *japonica* genomes, respectively. Interestingly, most of the selective‐sweep regions were clustered as genomic islands rather than randomly distributed across the *indica* and *japonica* genomes (Tables S8, S9). To assess whether these genomic islands contained known loci that control important agronomic traits, we compared their locations with previously mapped QTLs and cloned genes. We found a number of selective‐sweep regions overlapped with previously reported agronomic QTLs or contained cloned genes, such as *OsSDR* (Kim et al., 2009), *SSIIa* (Kawakatsu et al., 2009; Zhang et al., 2011), *OsPIN3t* (Miyashita et al., 2010; Wang et al., 2009; Zhang et al., 2012), and *OsLIC1* (Wang et al., 2008; Zhang et al., 2012), related to grain yield, eating quality, abiotic stress resistance, and plant type (Table S15). Furthermore, we found that these genes located in selective‐sweep regions were enriched in the grain yield category, followed by the abiotic stress resistance and plant‐type categories, while enrichment was quite low in the eating quality category (Figure 5). The results indicate yield and abiotic stress resistance traits have been frequently selected in rice landraces during the short‐term domestication. The reason is that the local farmers preferred not only for highly productive rice landraces but also for ones more resistant to tolerant of extreme environmental conditions. Interestingly, a number of genes, such as *FRRP1* (Flowering‐Related RING Protein 1) (Du et al., 2016), were also under selection during rice domestication from its wild ancestor as reported in a previous study (Xu et al., 2012). This suggests that a small number of genes with extremely large phenotypic effects have been targeted repeatedly by selection during domestication. Unexpectedly, we found only two common regions underwent selective sweeps in both *indica* and *japonica*, perhaps due to the divergence between the *indica* and *japonica* genomes. Additionally, we found multiple genes controlling the same traits, such as *UbL401* (Zhou et al., 2014) and *CYP703A3* (Yang et al., 2014) for sterility; *NLS1* (Tang et al., 2011) and *OsEP3A* (Singh, Giri, Singh, Siddiqui, & Nandi, 2013) for bacterial blight resistance; *AM1* (Sheng et al., 2014) and *OsPIN3t* (Miyashita et al., 2010; Wang et al., 2009; Zhang et al., 2012) for drought tolerance; and *OsHAK1* (Chen et al., 2015) and *OsHKT4* (Wang et al., 2015) for salt tolerance, in *indica* or *japo*nica selective‐sweep regions likely affected by short‐term domestication. This phenomenon showed signatures of parallel selection in the genomes of the two subspecies.

In summary, we provide a large dataset of genomic variation observed in on‐farm conserved rice landraces in this study. We identified millions of SNPs in representative rice landraces collected in 1980 and 2007, providing an unprecedented opportunity to finely resolve genome‐wide genetic diversity and selection footprints in landraces grown under on‐farm conservation conditions during a short‐term period of domestication. We demonstrated that farmer selection in combination with natural selection played an important role and strong selection can leave its footprint on genome‐wide polymorphism patterns. We propose that on‐farm conserved rice landraces during short‐term domestication had a highly polygenic basis with many loci responding to selection rather than a few loci with critical changes in response to selection. Moreover, the loci affecting important agronomic traits and biotic or abiotic stress response were particularly targeted. Our integrative analyses demonstrate that the rice landraces grown under on‐farm conservation conditions have the potential to be a dynamic, evolving genetic system that can undergo continuous differentiation and variation in response to evolutionary pressures, both natural and those imposed by farmers. On‐farm, in situ conservation is a recommended strategy to conserve crop genetic resources.

## Data Availability

All genome sequence data have been deposited in the NCBI Sequence Read Archive (SRA) under project Accession Number: PRJNA342109.
