# Supplementary material for: Genomic analyses reveal selection footprints in rice landraces grown under on‐farm conservation conditions during a short‐term period of domestication
Source: Evol Appl. 2019 Sep 30;13(2):290–302. doi: 10.1111/eva.12866 (PMC6976955; doi:10.1111/eva.12866)
Supplement: Supplementary file 2 [file EVA-13-290-s002.pdf]

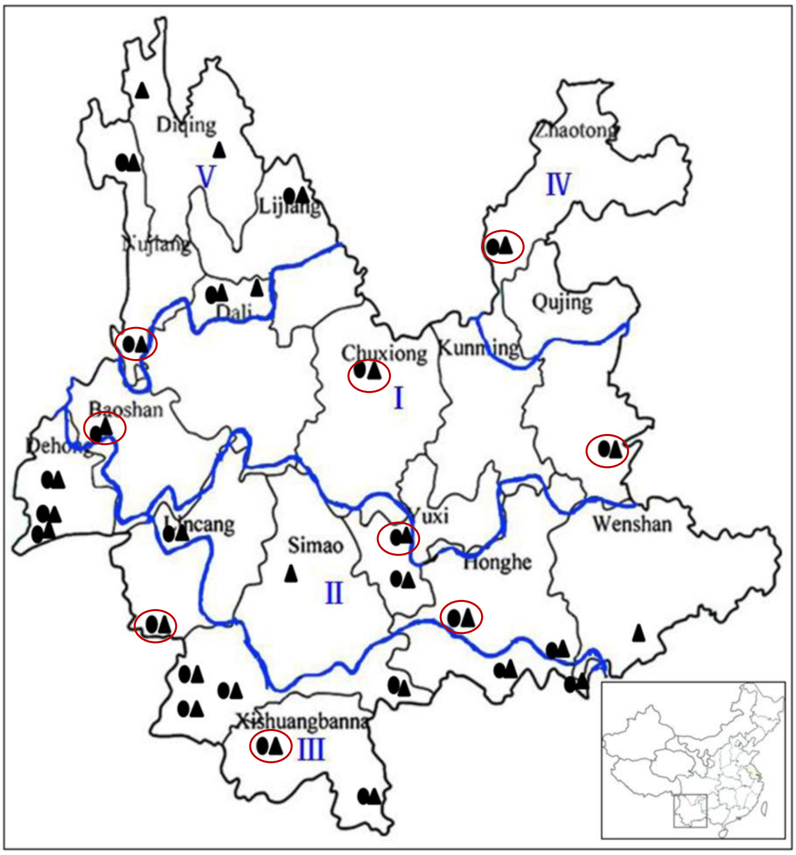

Figure S1. Geographical distribution of the collected rice landraces. To represent as much the genetic diversity and wide geographic distribution of rice landraces in Yunnan as possible, we first carefully selected 600 rice landraces from 32 counties (including 332 accessions collected in 1980, and 268 accessions collected in 2007), covering a wide geographic distribution and diverse growing conditions. Solid circles and triangles represent the rice landraces in 1980 and 2007, respectively. Further, the core collection (108 accessions, including 56 accessions collected in 1980, and 52 accessions collected in 2007) was sampled for whole-genome sequencing in this study from the base collection (600 accessions), which are marked with a red circle. I, Indica/Japonica rice ecological zone at the middle of Yunnan; II, Single/double-season rice ecological zone of southern Yunnan; III, Lowland/upland-rice ecological zone at the edge of southern Yunnan; IV, Japonica-rice ecological zone on the plateau of northeast Yunnan; V, Cold Japonica-rice ecological zone of northwest Yunnan.

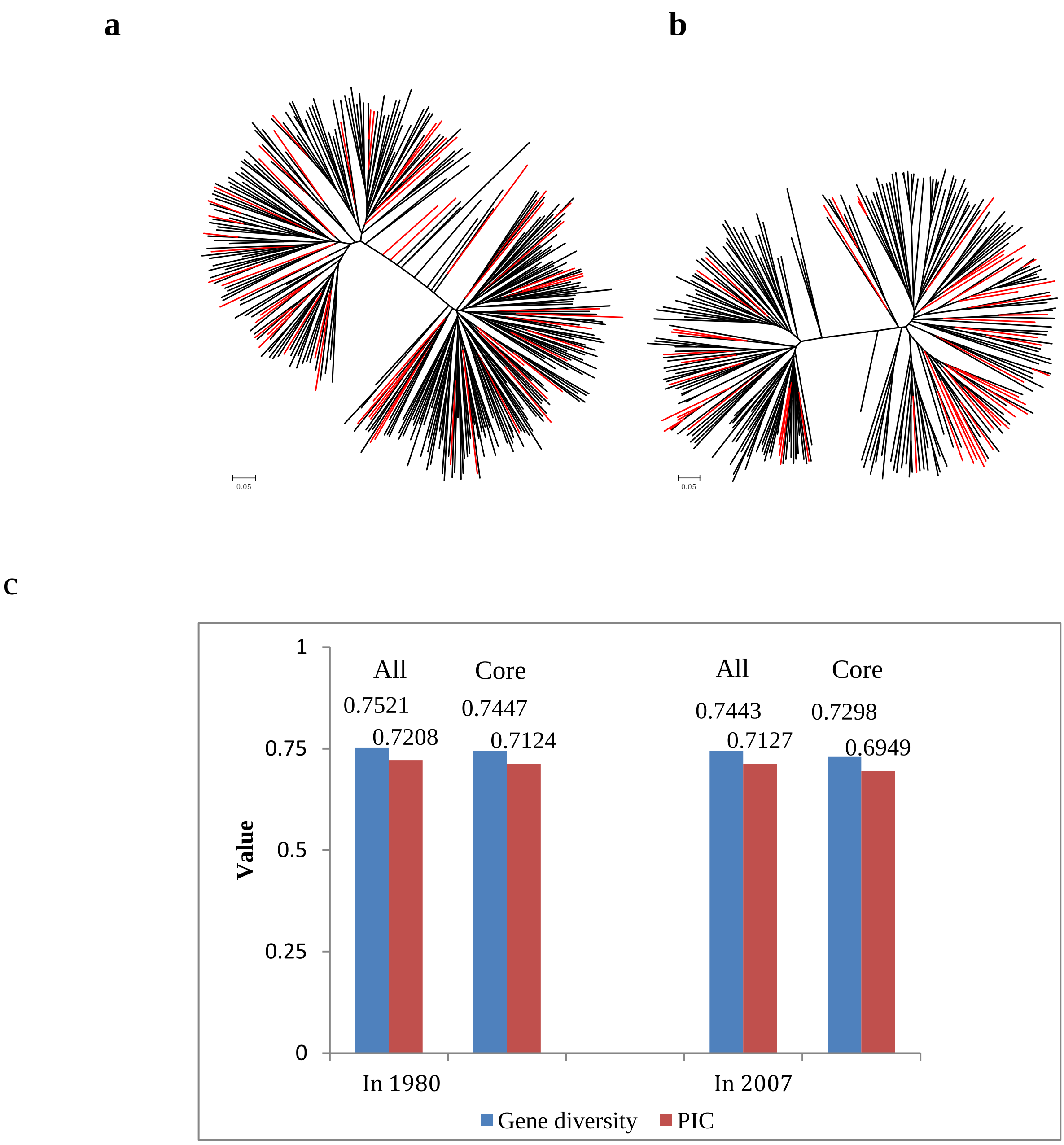

Figure S2. The neighbor joining trees based on genetic distances of 332 rice accessions collected in 1980 (a) and 268 accessions collected in 2007 (b) calculated using 48 SSR markers. (c) Comparison of genetic diversity between the subset of accessions and all accessions.

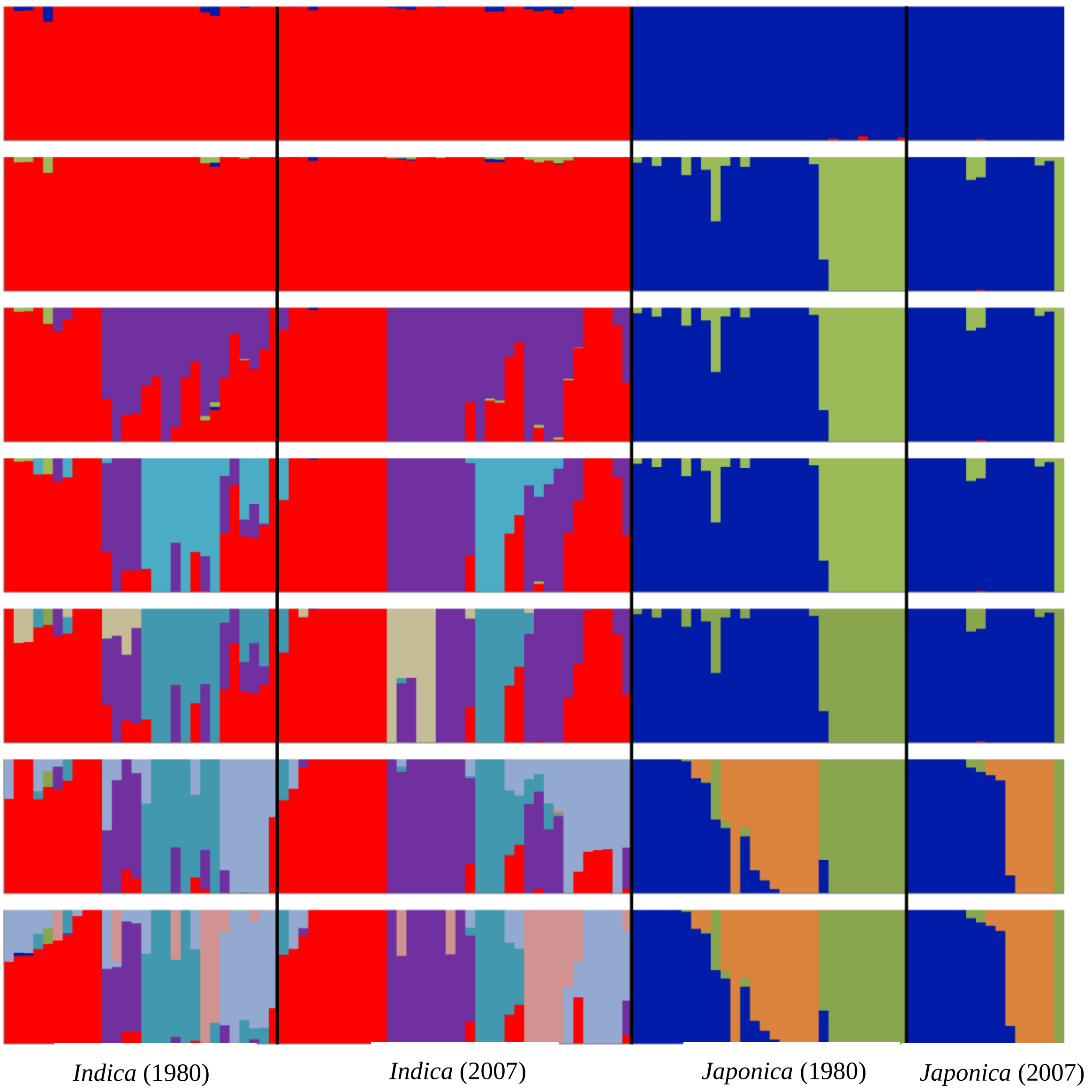

Figure S3. Population structure analysis using ADMIXTURE. Each color represents one population. Each accession is represented by a vertical bar, and the length of each colored segment in each vertical bar represents the proportion contributed by ancestral populations.

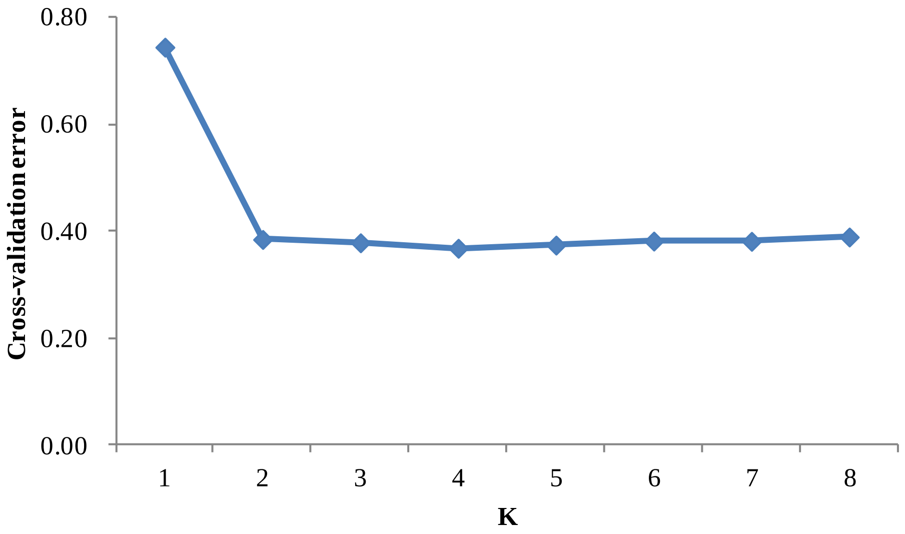

Figure S4. The cross-validation error for each given k. The optimal k value was 2 because of a low cross-validation error when k = 2.

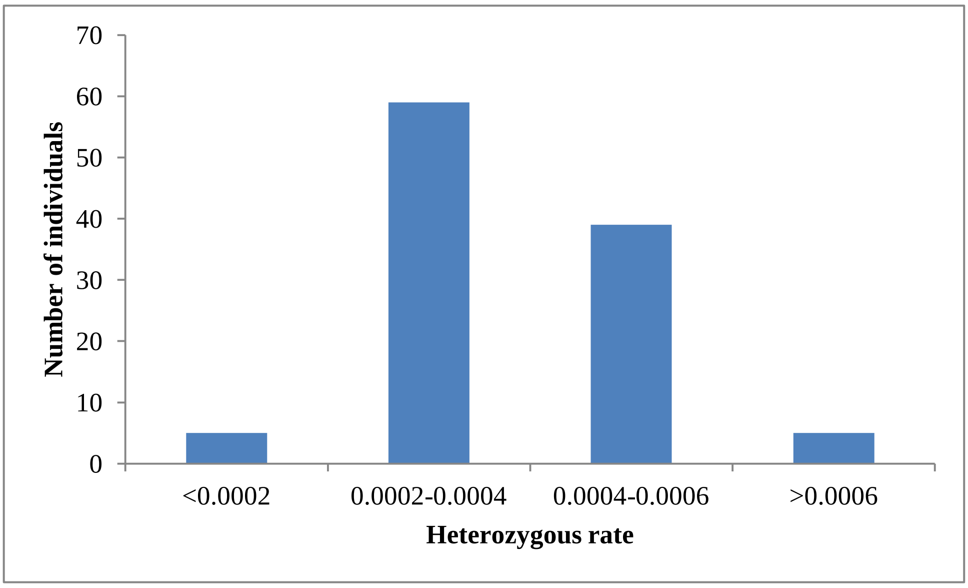

Figure S5. Heterozygous rate of SNPs in all rice landraces.

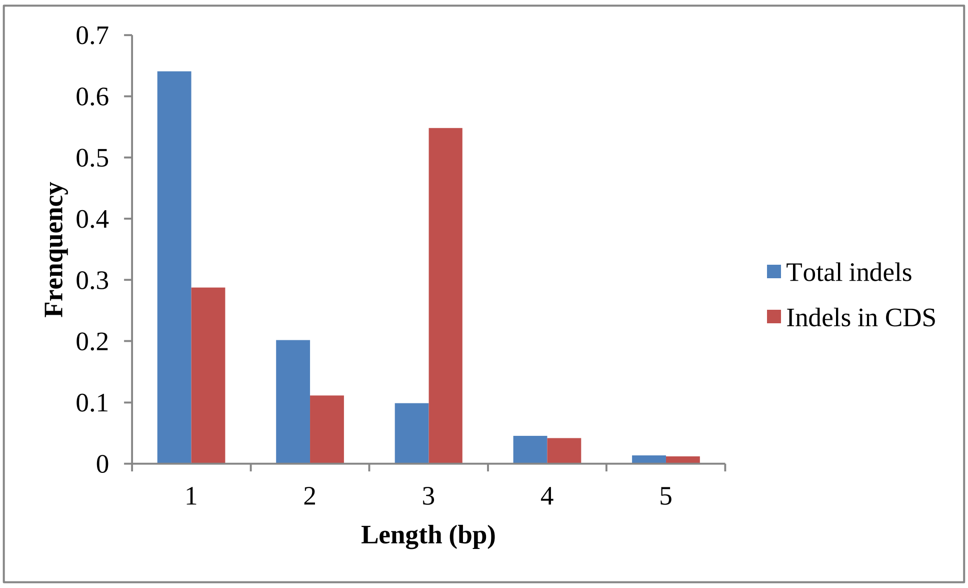

Figure S6. Length distribution of indels. Length distribution of all the indels and indels in coding sequences was plotted. Of all the indels, the longer the indel is, the less would it be in the genome. Of the indels in CDS, 3 bp indels which would not cause frameshift are the most common.

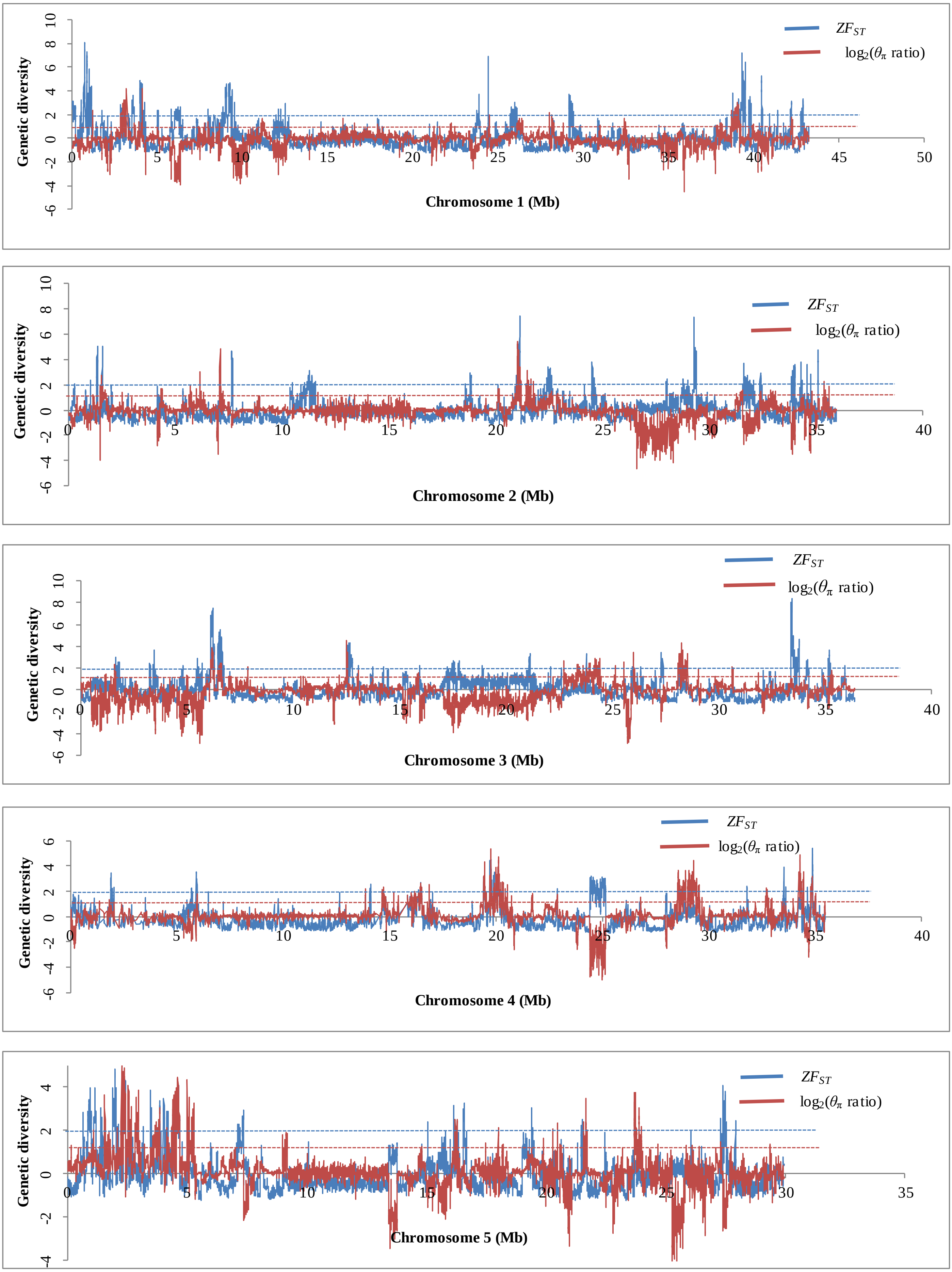

ZF_{ST}
$\log_2(\theta_\pi$ ratio)
Genetic diversity
Chromosome 1 (Mb)
Chromosome 2 (Mb)
Chromosome 3 (Mb)
Chromosome 4 (Mb)
Chromosome 5 (Mb)

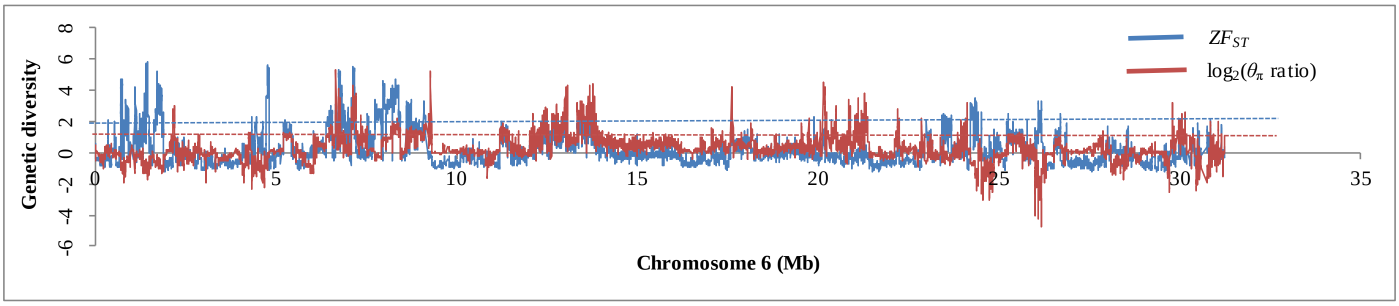
Genetic diversity
ZF_{ST}
$\log_2(\theta_\pi$ ratio)
Chromosome 6 (Mb)

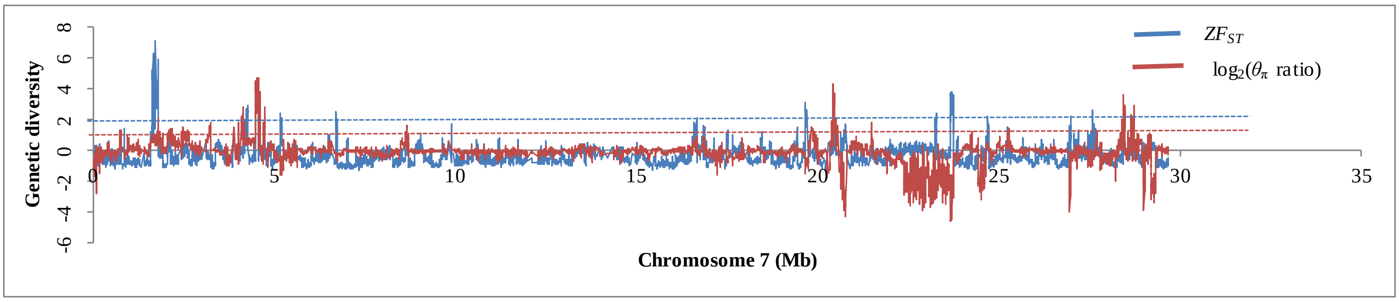
Genetic diversity
ZF_{ST}
$\log_2(\theta_\pi$ ratio)
Chromosome 7 (Mb)

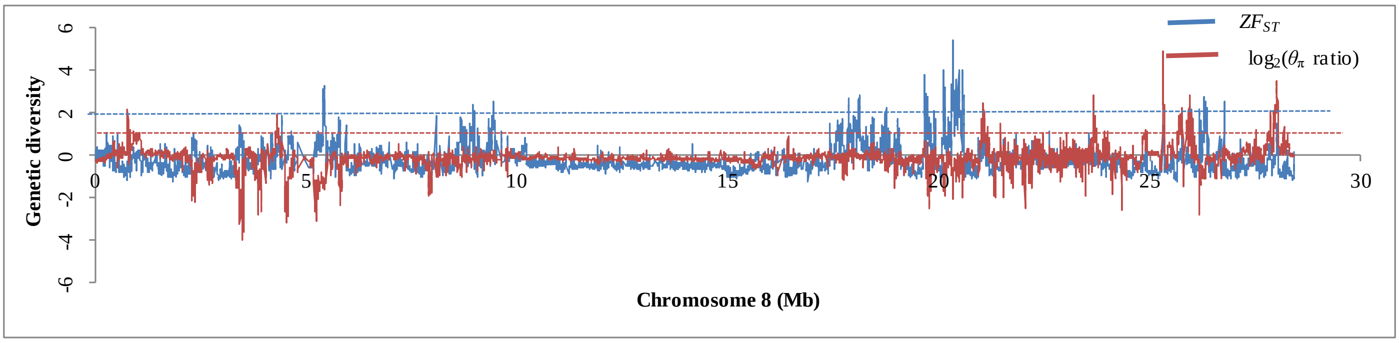
Genetic diversity
ZF_{ST}
$\log_2(\theta_\pi$ ratio)
Chromosome 8 (Mb)

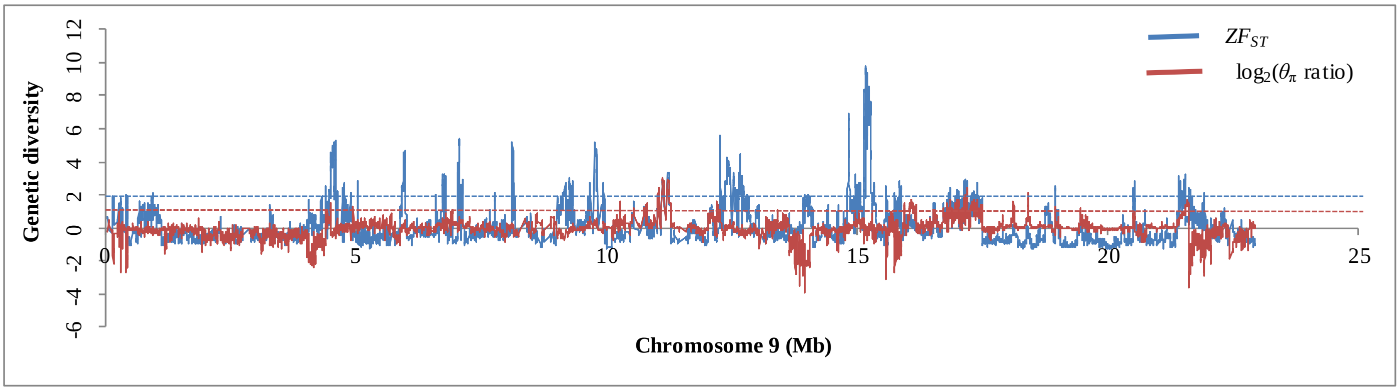
Genetic diversity
ZF_{ST}
$\log_2(\theta_\pi$ ratio)
Chromosome 9 (Mb)

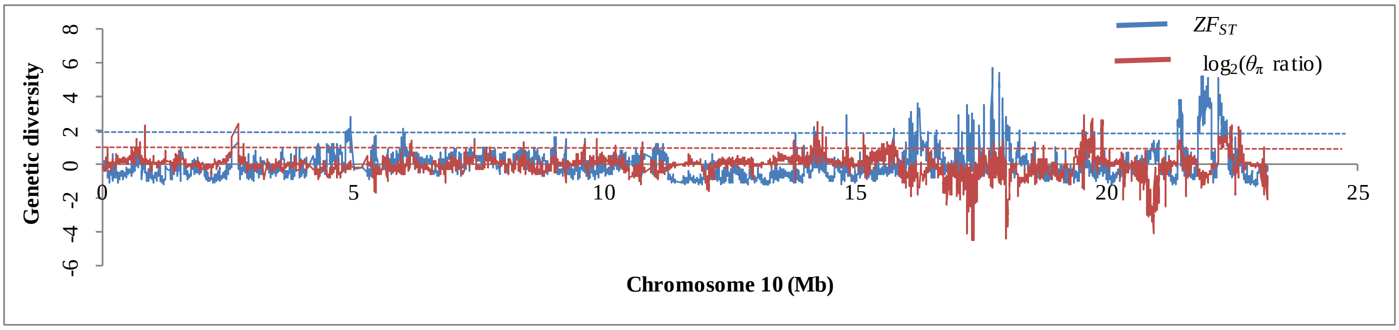
Genetic diversity
ZF_{ST}
$\log_2(\theta_\pi$ ratio)
Chromosome 10 (Mb)

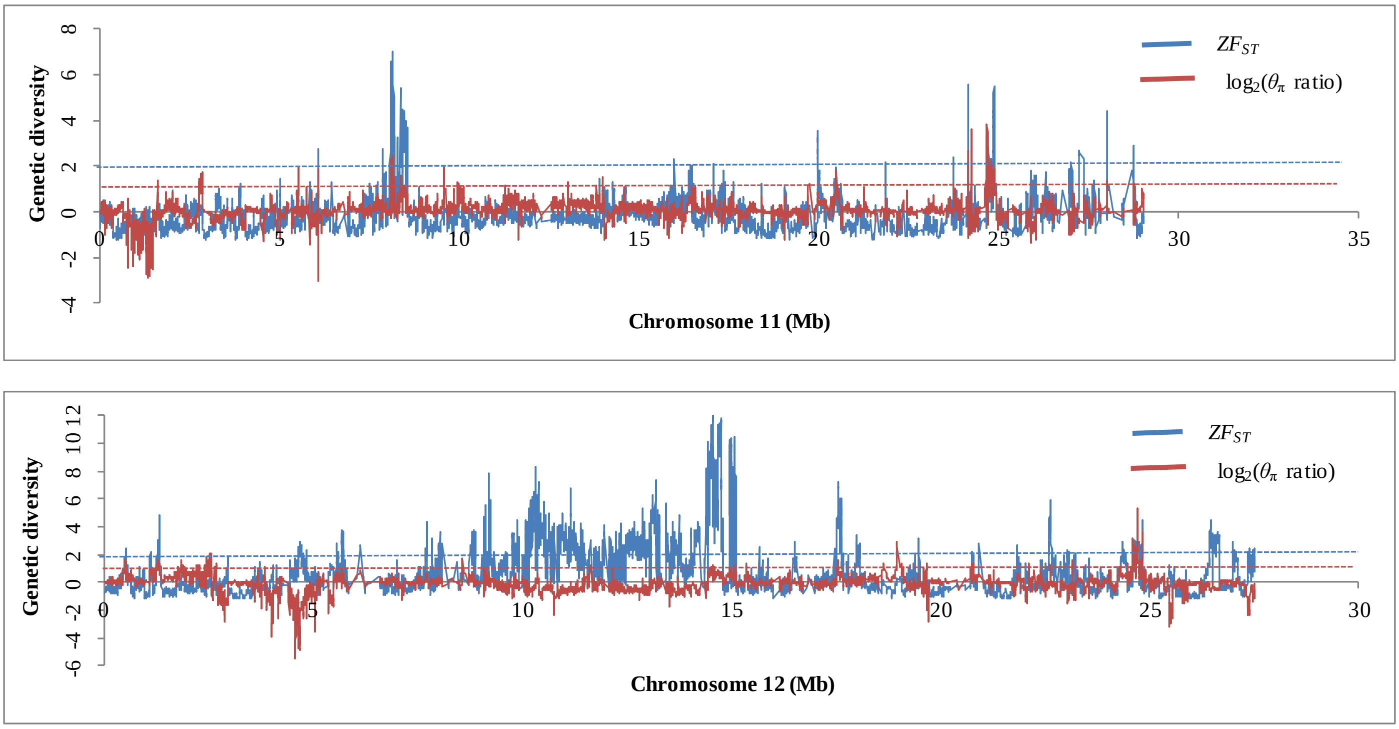

Figure S7. Genetic diversity of chromosome 1–12 of *indica* rice landraces.

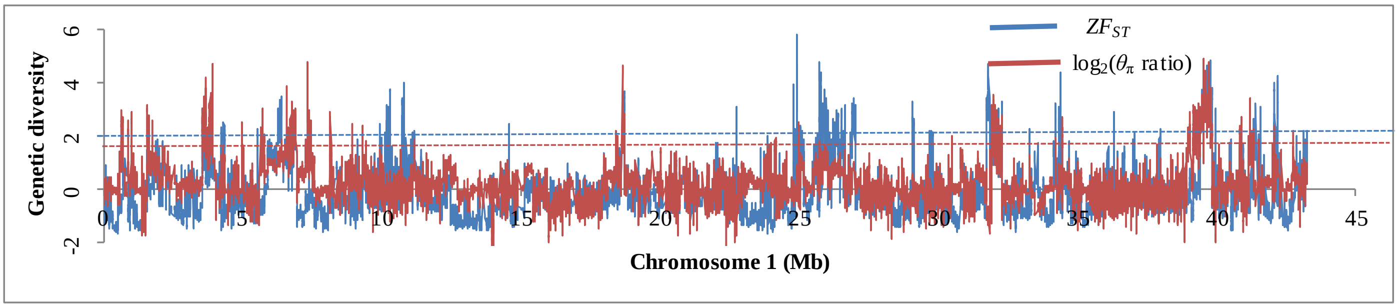

ZF_{ST}
$\log_2(\theta_\pi$ ratio)
Genetic diversity
-2
0
2
4
6
0
5
10
15
20
25
30
35
40
45
Chromosome 1 (Mb)

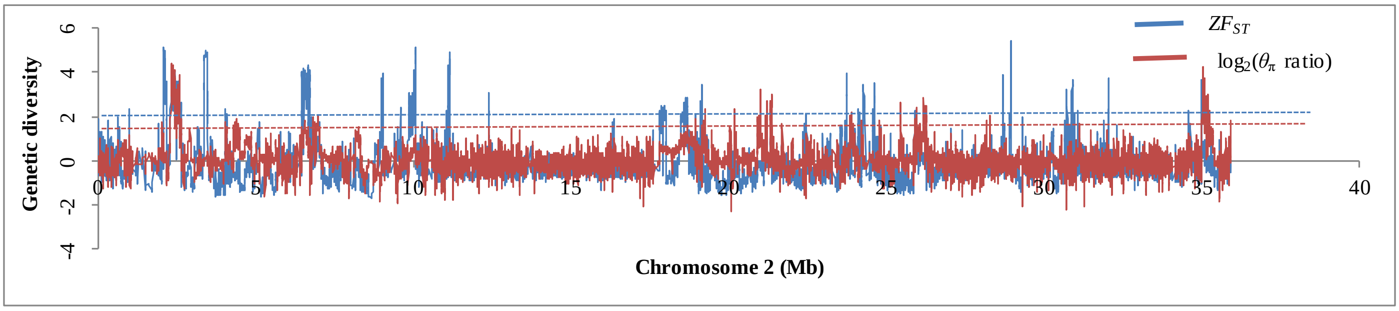

ZF_{ST}
$\log_2(\theta_\pi$ ratio)
Genetic diversity
-4
-2
0
2
4
6
0
5
10
15
20
25
30
35
40
Chromosome 2 (Mb)

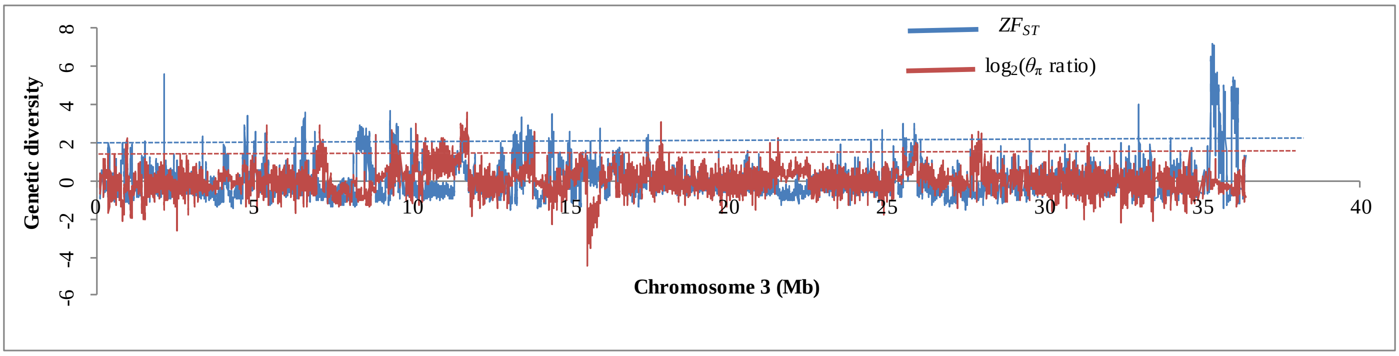

ZF_{ST}
$\log_2(\theta_\pi$ ratio)
Genetic diversity
-6
-4
-2
0
2
4
6
8
0
5
10
15
20
25
30
35
40
Chromosome 3 (Mb)

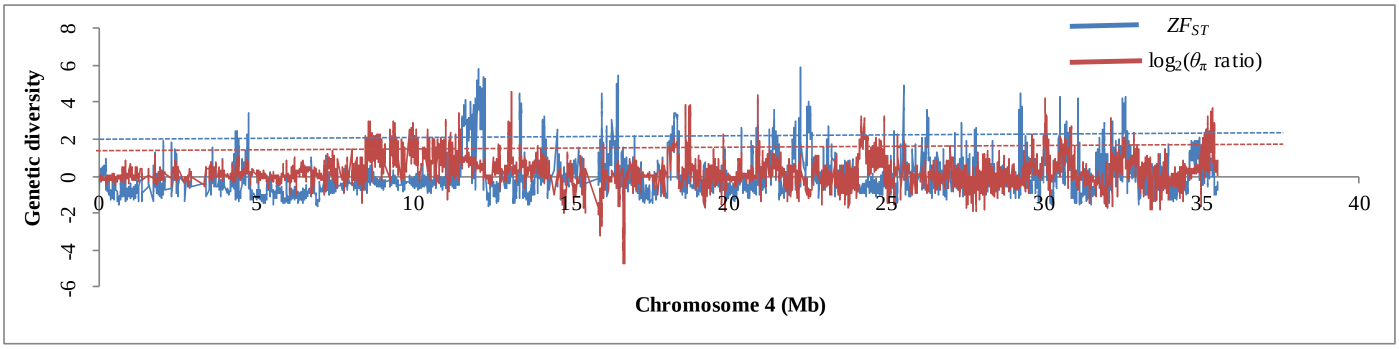

ZF_{ST}
$\log_2(\theta_\pi$ ratio)
Genetic diversity
-6
-4
-2
0
2
4
6
8
0
5
10
15
20
25
30
35
40
Chromosome 4 (Mb)

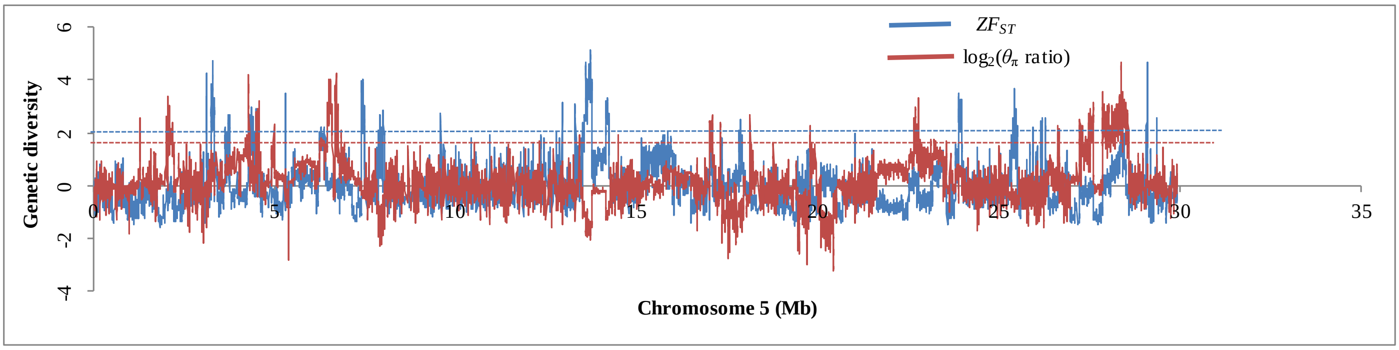

ZF_{ST}
$\log_2(\theta_\pi$ ratio)
Genetic diversity
-4
-2
0
2
4
6
0
5
10
15
20
25
30
35
Chromosome 5 (Mb)

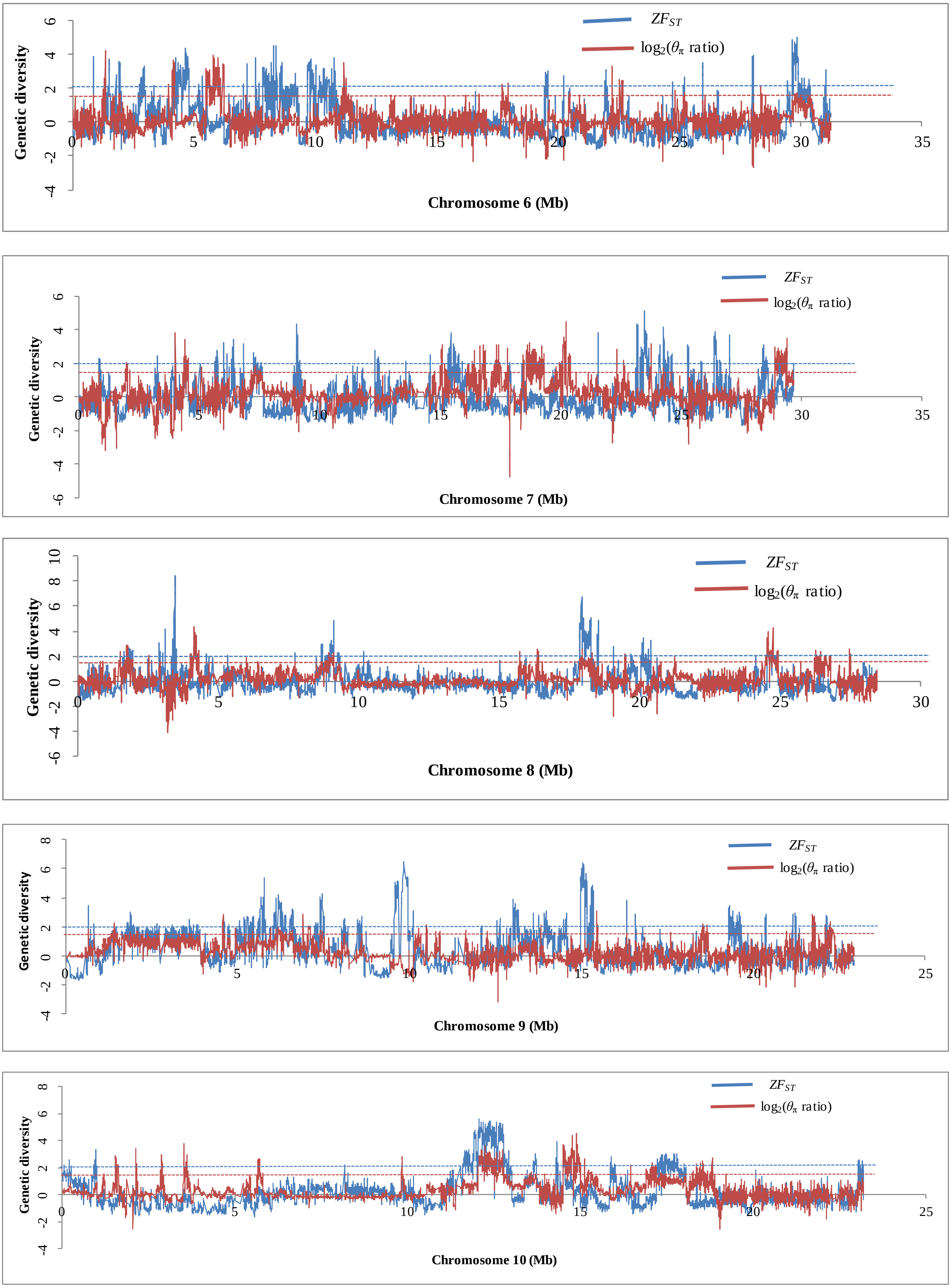

ZFST
log2(θπ ratio)
Genetic diversity
Chromosome 6 (Mb)
ZFST
log2(θπ ratio)
Genetic diversity
Chromosome 7 (Mb)
ZFST
log2(θπ ratio)
Genetic diversity
Chromosome 8 (Mb)
ZFST
log2(θπ ratio)
Genetic diversity
Chromosome 9 (Mb)
ZFST
log2(θπ ratio)
Genetic diversity
Chromosome 10 (Mb)

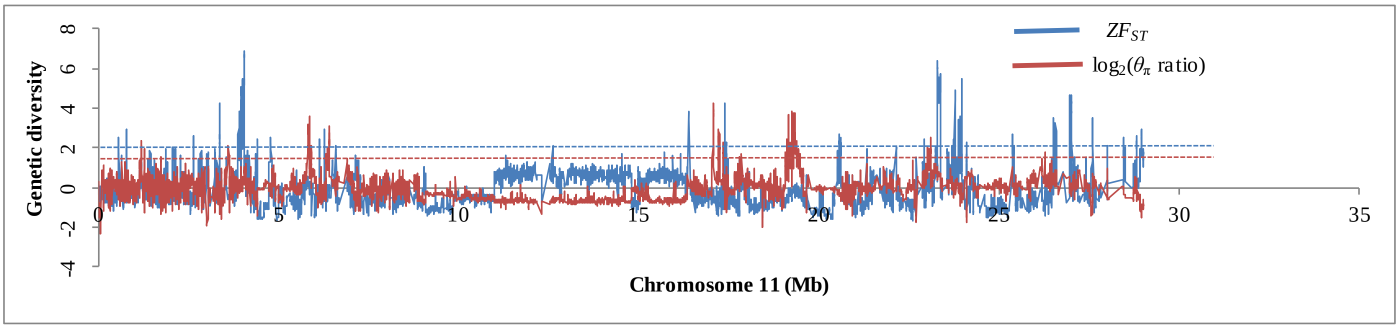

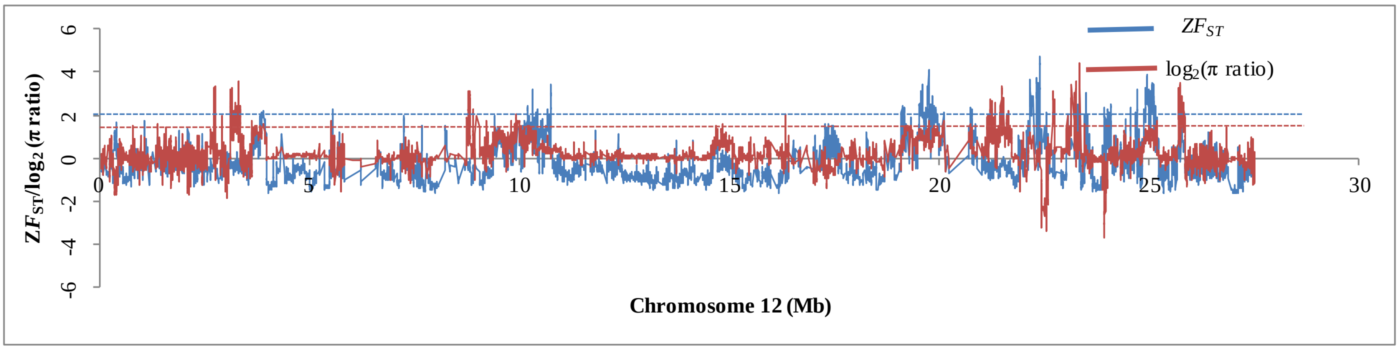

Figure S8. Genetic diversity of chromosome 1–12 of *japonica* rice landraces.

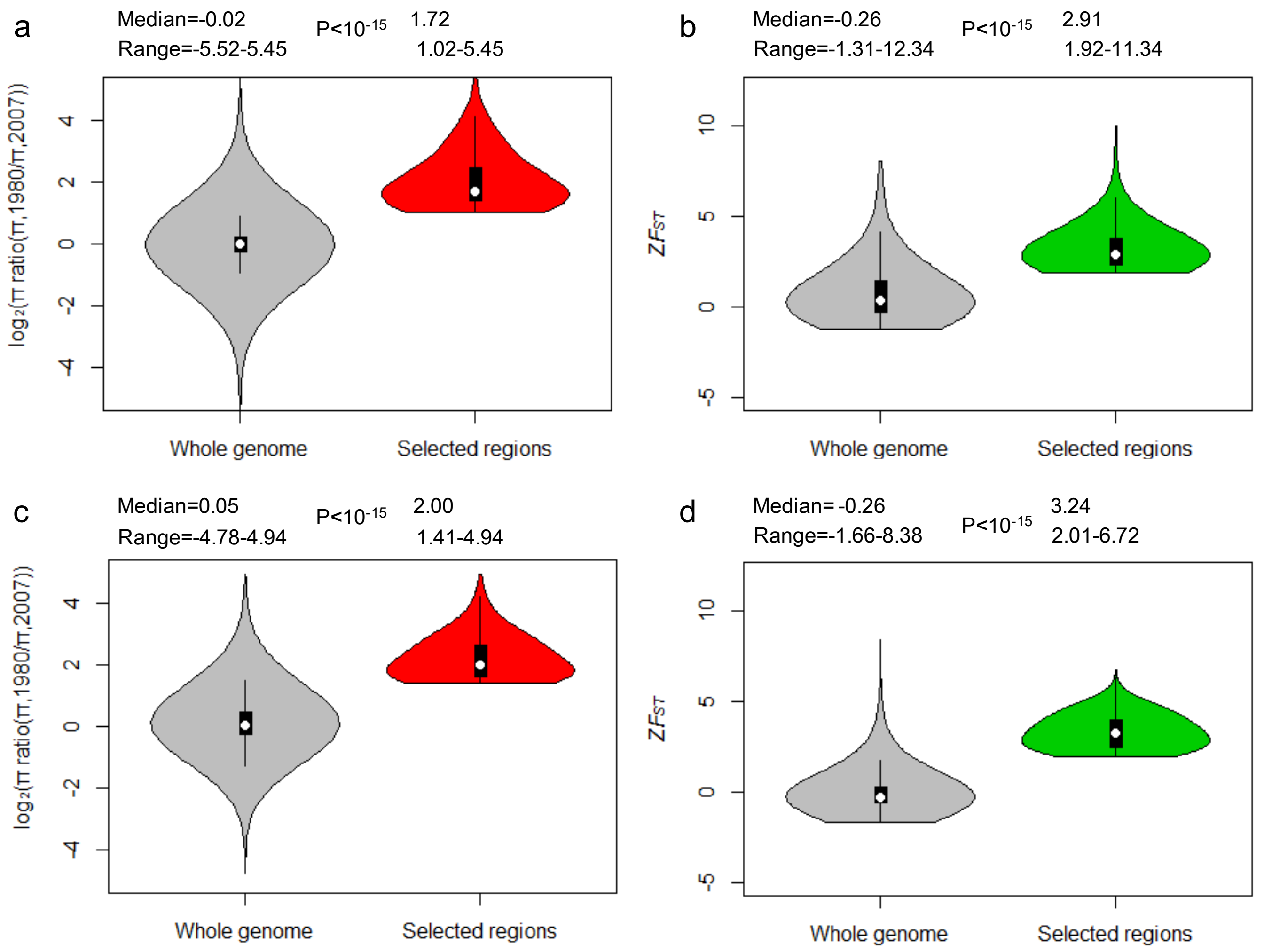

Figure S9. Violin plot of log2 (θπ ratio) and ZF_{ST} values for regions of *indica* (a, b) and *japonica* (c, d) rice landraces that have undergone selection versus the whole genome regions. Each ‘‘violin’’ with the width depicting a 90 °-rotated kernel density trace and its reflection. Vertical black boxes denote the interquartile range (IQR) between the first and third quartiles (25th and 75th percentiles, respectively) and the white point inside denotes the median. Vertical black lines denote the lowest and highest values within 1.5 times IQR from the first and third quartiles, respectively. The statistical significance was calculated by the Mann-Whitney U test.

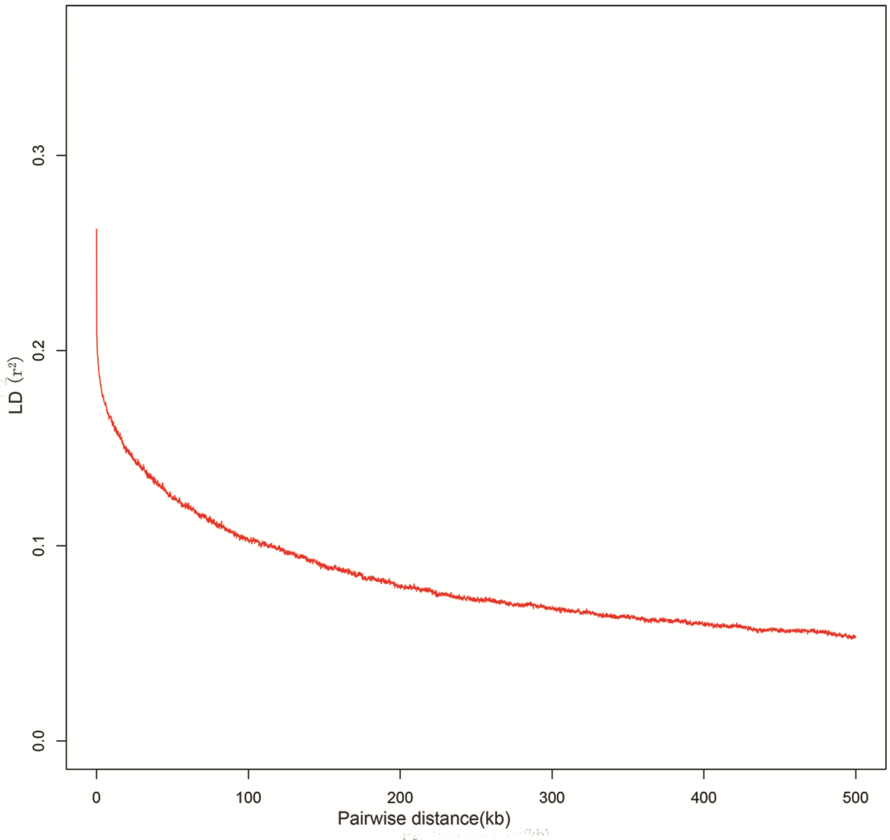

Figure S10. Linkage disequilibrium (LD) decays within 100 kb for rice landraces.

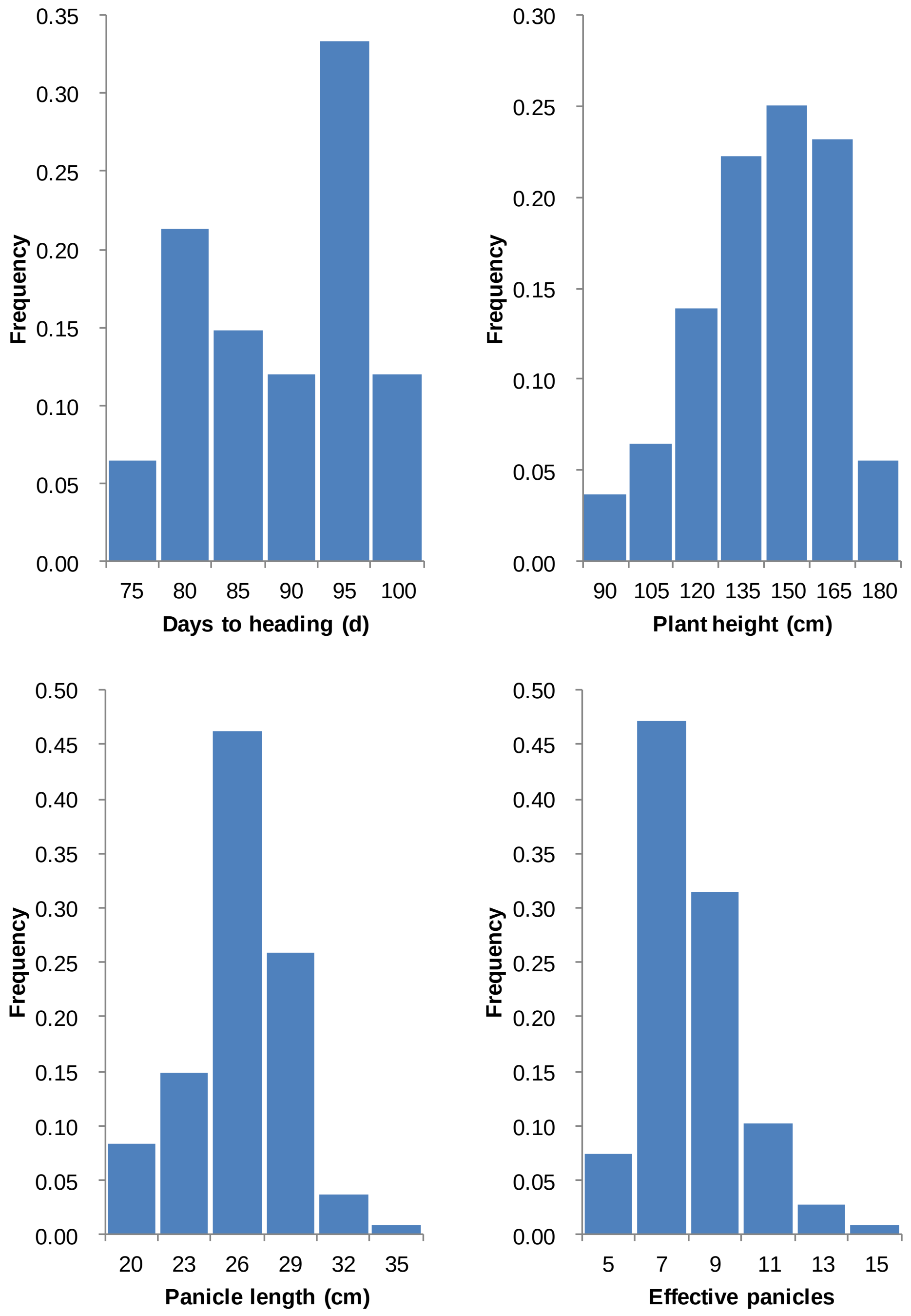

Frequency
0.35
0.30
0.25
0.20
0.15
0.10
0.05
0.00
75 80 85 90 95 100
Days to heading (d)
Frequency
0.30
0.25
0.20
0.15
0.10
0.05
0.00
90 105 120 135 150 165 180
Plant height (cm)
Frequency
0.50
0.45
0.40
0.35
0.30
0.25
0.20
0.15
0.10
0.05
0.00
20 23 26 29 32 35
Panicle length (cm)
Frequency
0.50
0.45
0.40
0.35
0.30
0.25
0.20
0.15
0.10
0.05
0.00
5 7 9 11 13 15
Effective panicles

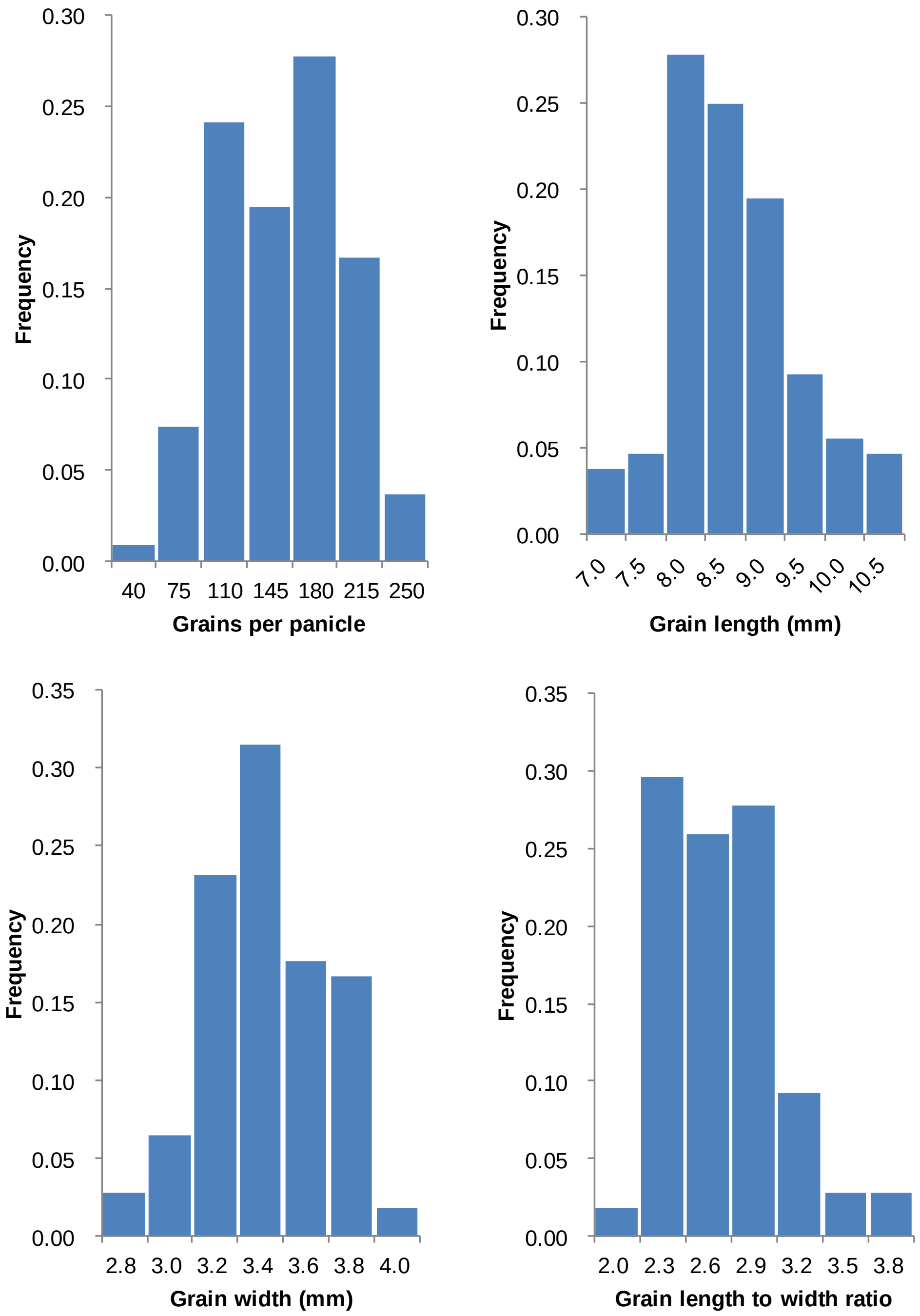
Frequency
0.30
0.25
0.20
0.15
0.10
0.05
0.00
40 75 110 145 180 215 250
Grains per panicle
Frequency
0.30
0.25
0.20
0.15
0.10
0.05
0.00
7.0 7.5 8.0 8.5 9.0 9.5 10.0 10.5
Grain length (mm)
Frequency
0.35
0.30
0.25
0.20
0.15
0.10
0.05
0.00
2.8 3.0 3.2 3.4 3.6 3.8 4.0
Grain width (mm)
Frequency
0.35
0.30
0.25
0.20
0.15
0.10
0.05
0.00
2.0 2.3 2.6 2.9 3.2 3.5 3.8
Grain length to width ratio

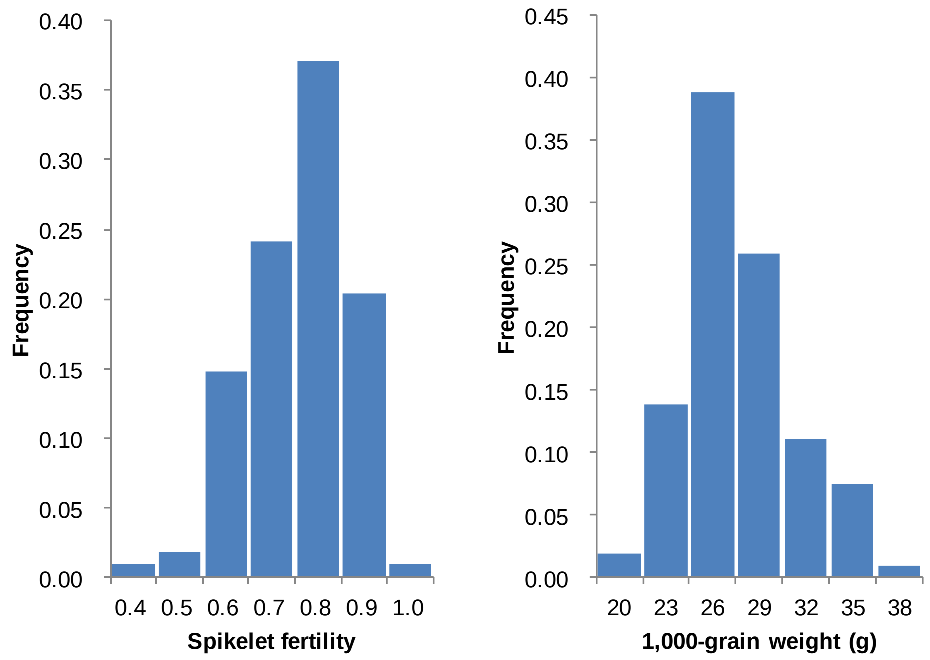

Figure S11. Frequency distribution of variation in the agronomic traits.

a

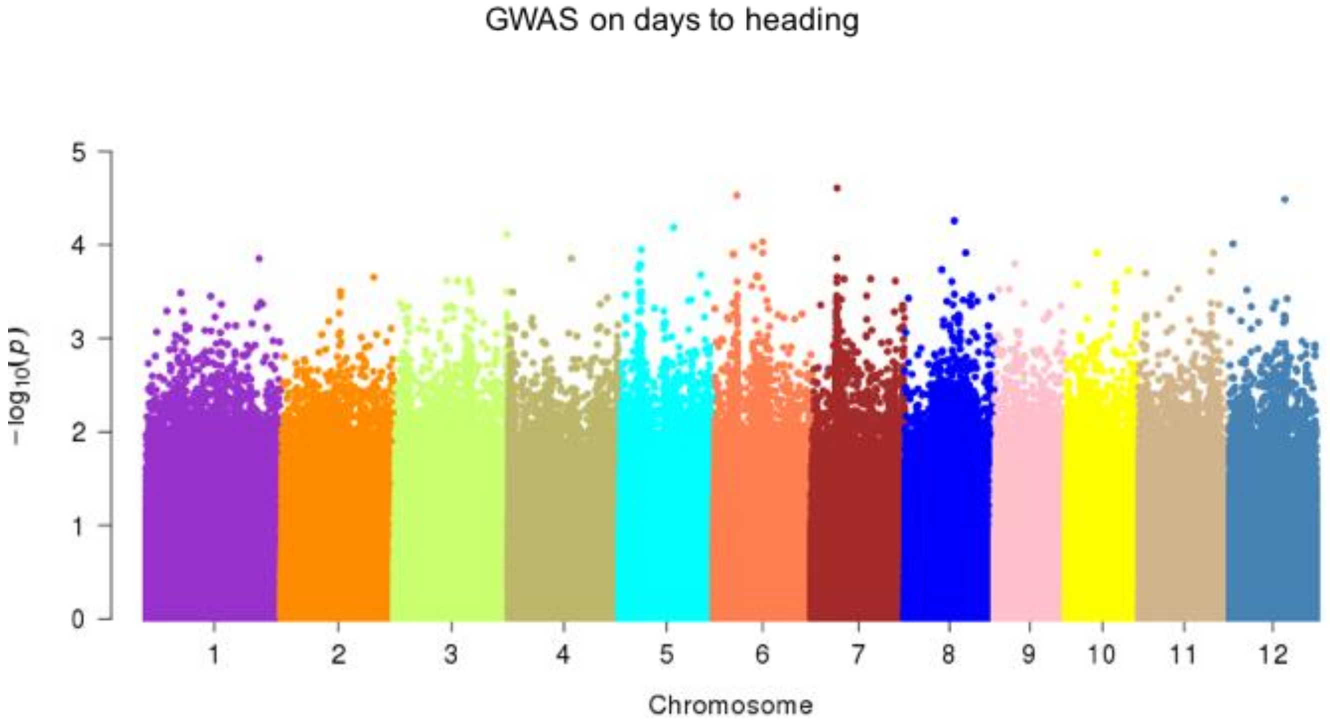

GWAS on days to heading
5
4
3
2
1
0
−log10(p)
1
2
3
4
5
6
7
8
9
10
11
12
Chromosome

b

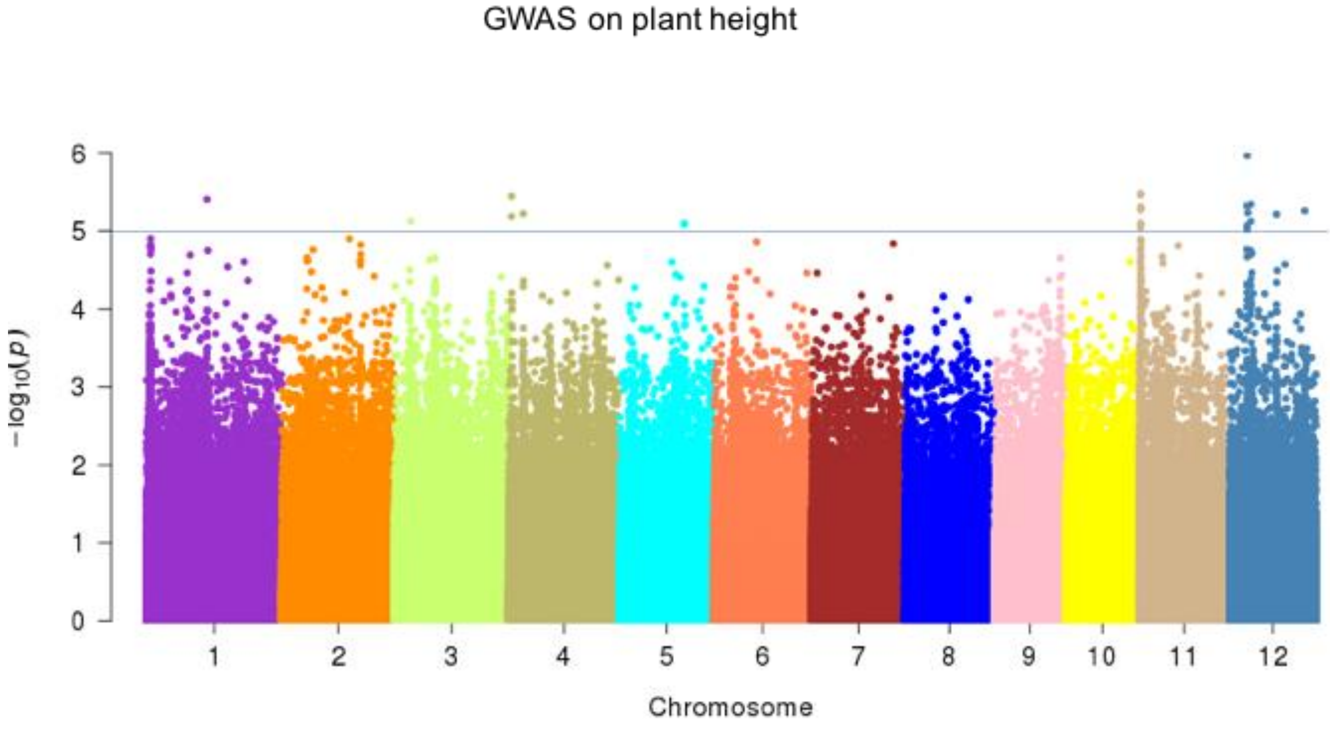

GWAS on plant height
6
5
4
3
2
1
0
−log10(p)
1
2
3
4
5
6
7
8
9
10
11
12
Chromosome

c

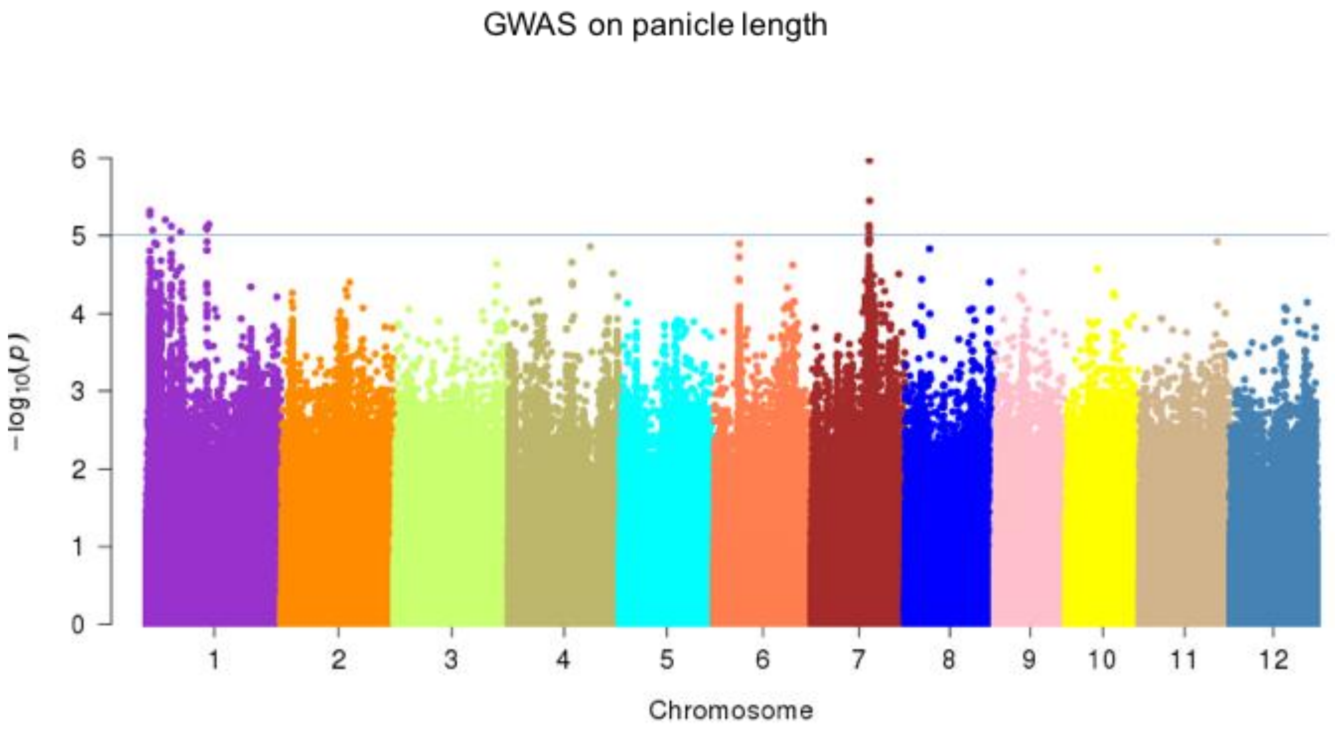

GWAS on panicle length
−log10(p)
0
1
2
3
4
5
6
1
2
3
4
5
6
7
8
9
10
11
12
Chromosome

d

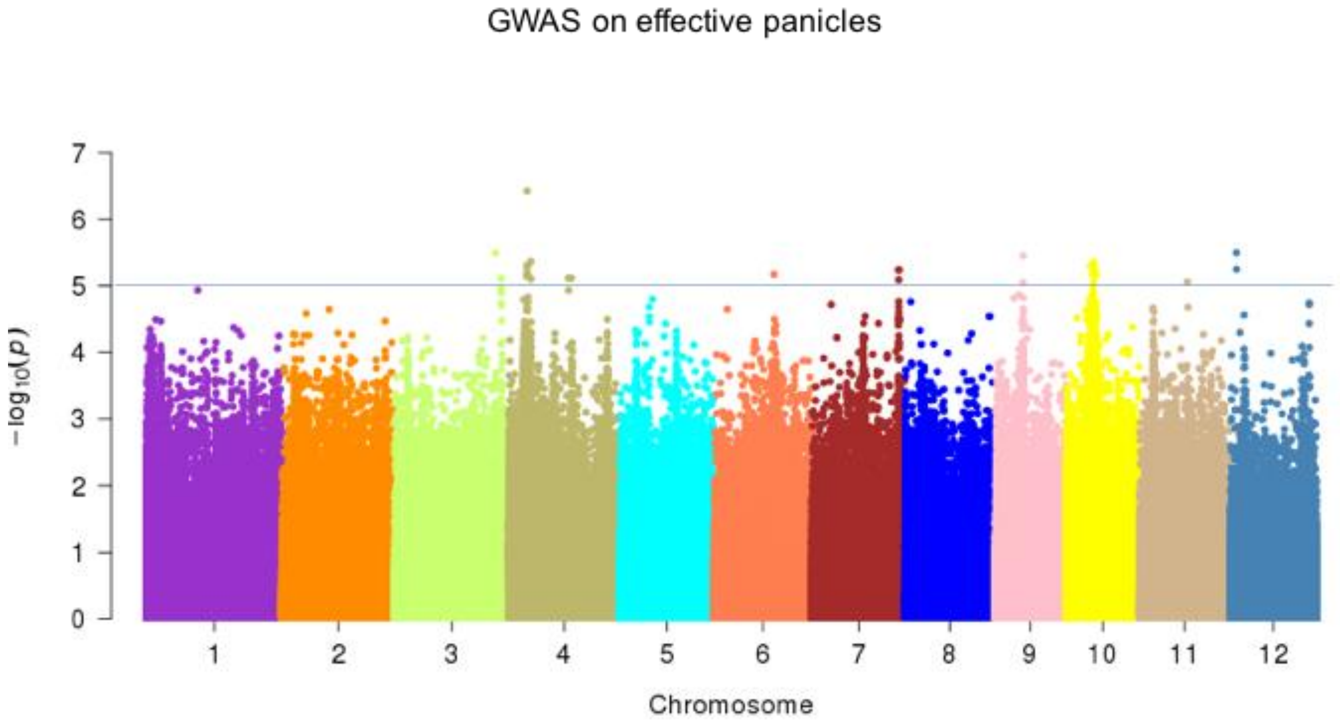

GWAS on effective panicles
−log10(p)
0
1
2
3
4
5
6
7
1
2
3
4
5
6
7
8
9
10
11
12
Chromosome

e

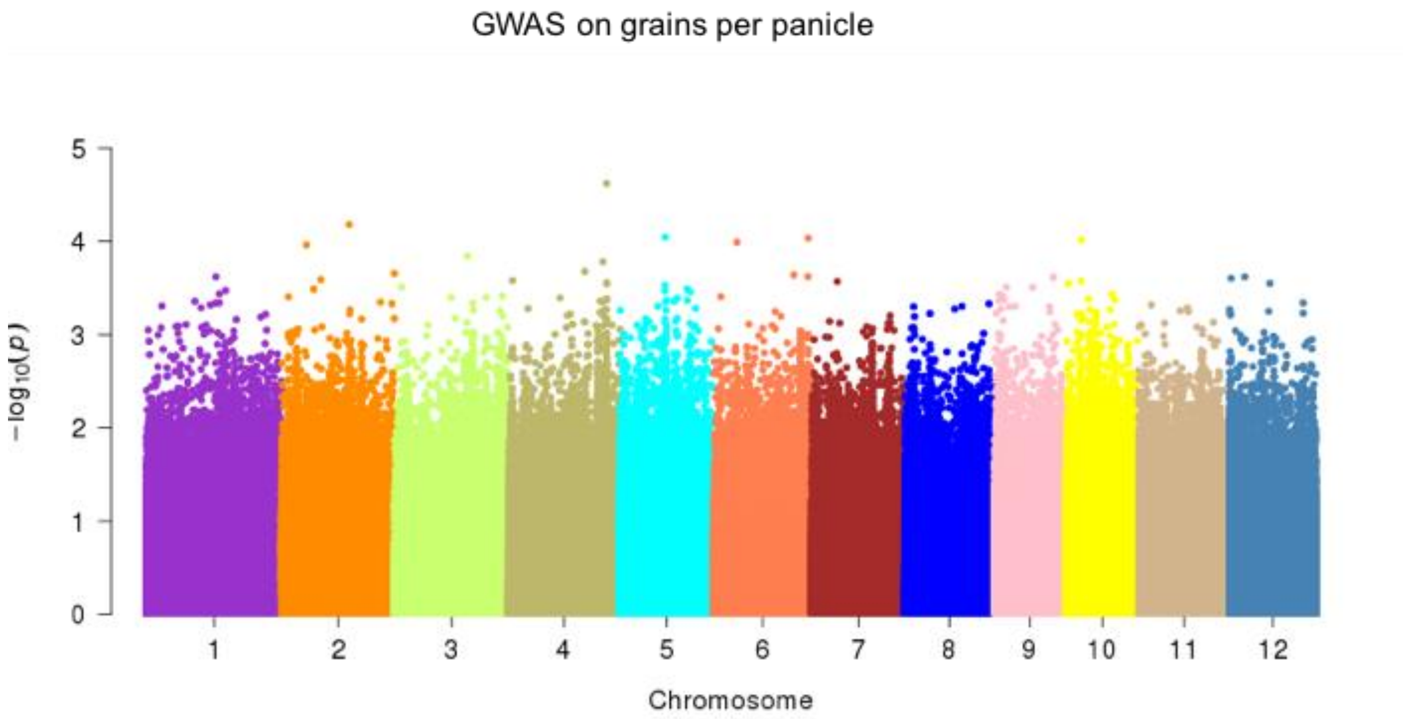
GWAS on grains per panicle
5
4
3
2
1
0
−log10(p)
1 2 3 4 5 6 7 8 9 10 11 12
Chromosome

f

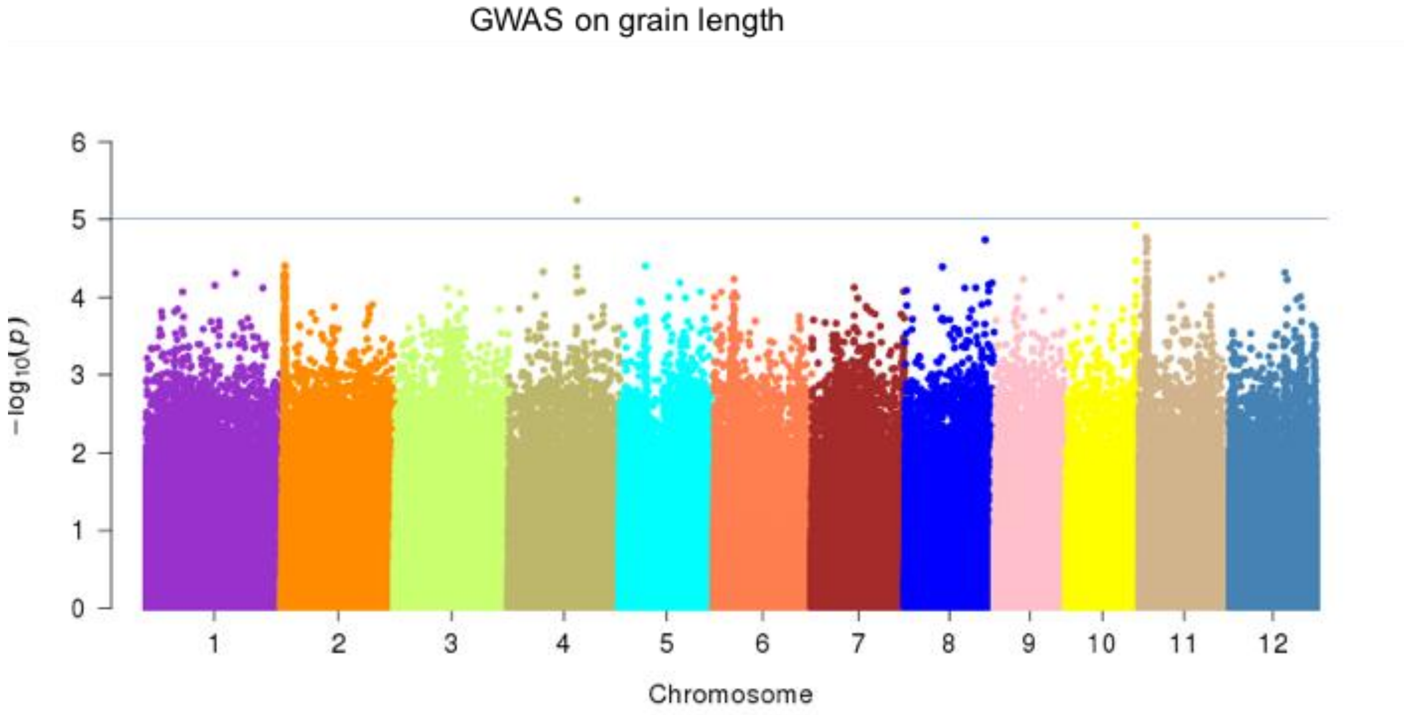
GWAS on grain length
6
5
4
3
2
1
0
−log10(p)
1 2 3 4 5 6 7 8 9 10 11 12
Chromosome

g

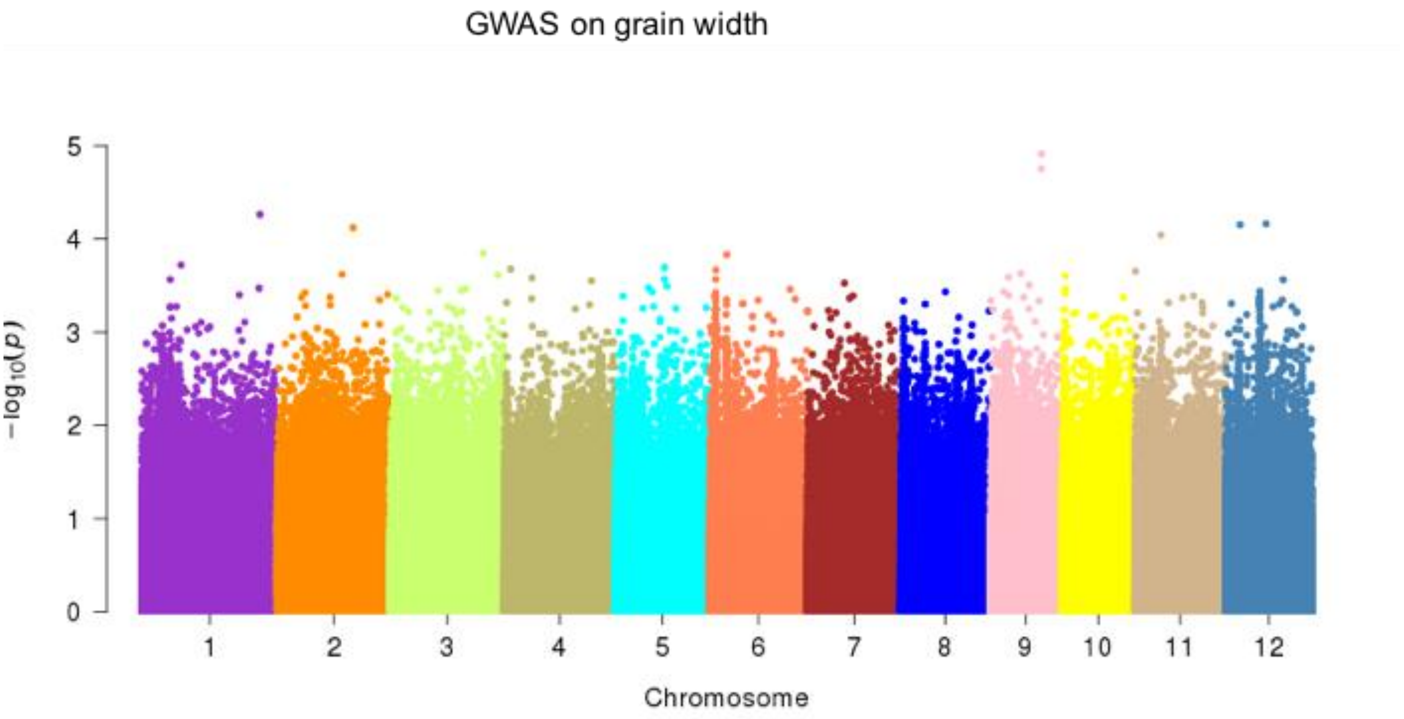
GWAS on grain width
5
4
3
2
1
0
$-\log_{10}(p)$
1
2
3
4
5
6
7
8
9
10
11
12
Chromosome

h

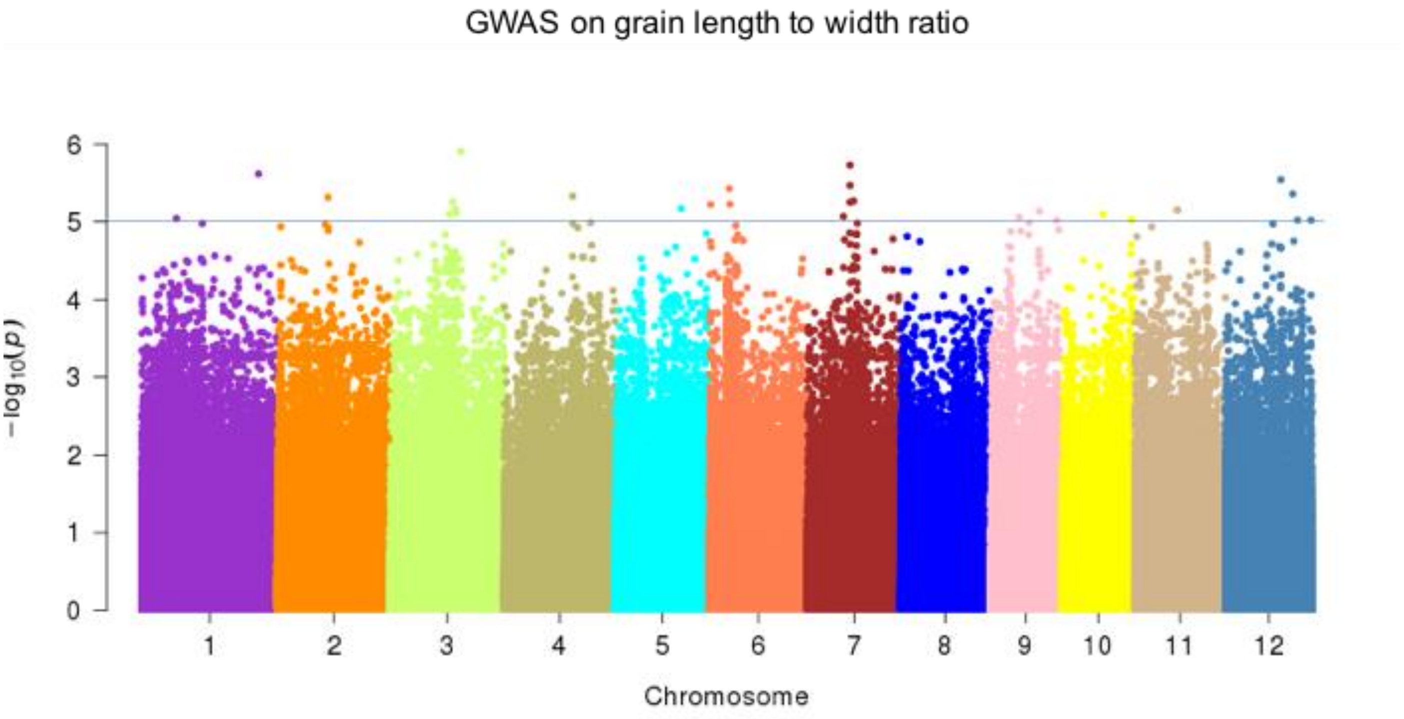
GWAS on grain length to width ratio
6
5
4
3
2
1
0
$-\log_{10}(p)$
1
2
3
4
5
6
7
8
9
10
11
12
Chromosome

i

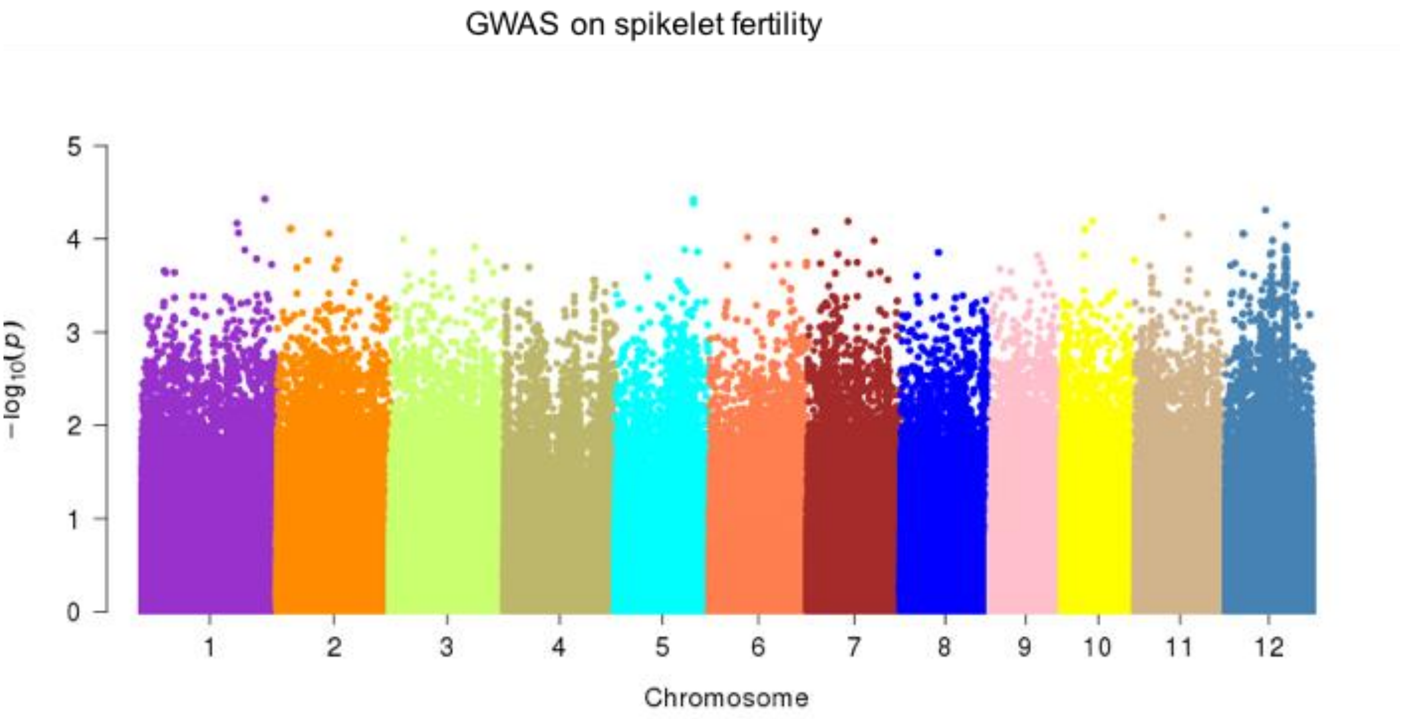

j

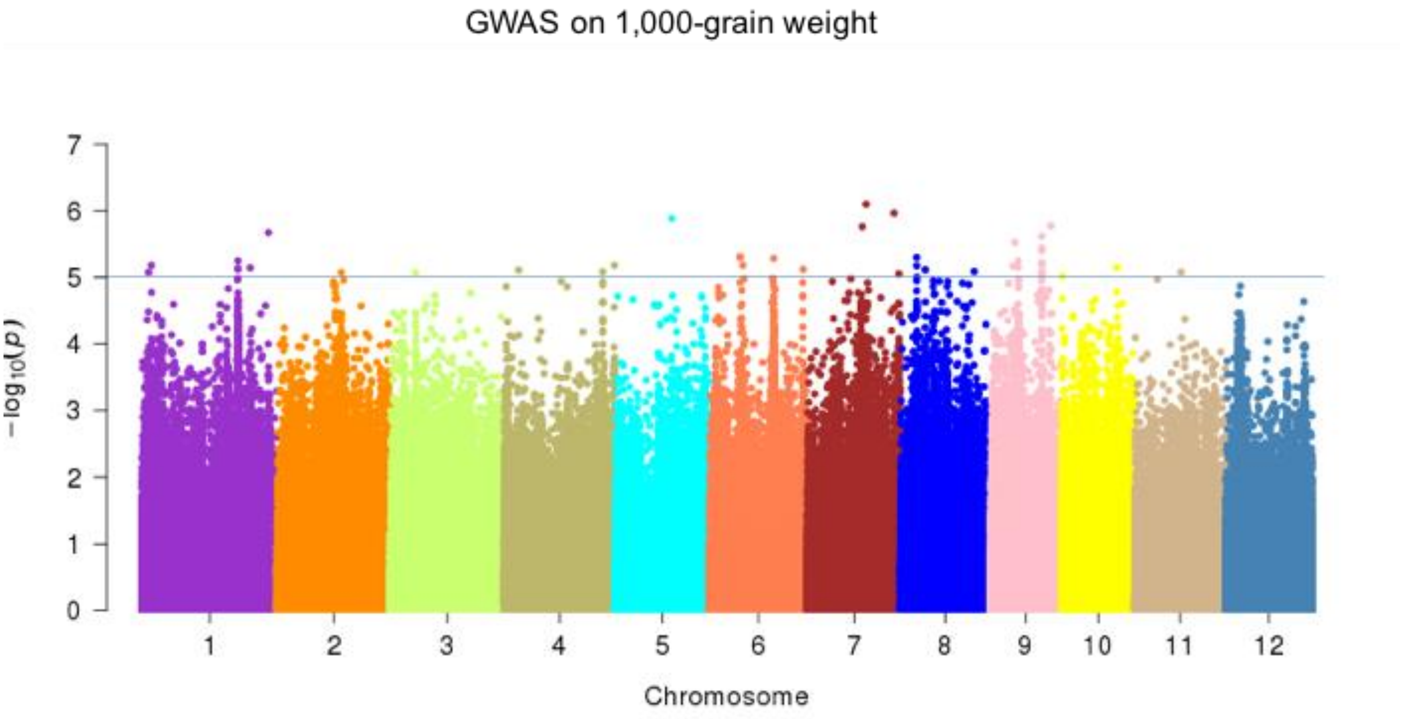

Figure S12. Genome-wide association study of ten agronomic traits. Manhattan plots for days to heading (a), plant height (b), panicle length (c), effective panicles (d), grains per panicle (e), grain length (f), grain width (g), grain length to width ratio (h), spikelet fertility (i), and 1,000-grain weight (j). The genome-wide significance threshold is shown as a line.
